# Do sounds near the hand facilitate tactile reaction times? Four experiments and a meta-analysis provide mixed support and suggest a small effect size

**DOI:** 10.1007/s00221-020-05771-5

**Published:** 2020-03-19

**Authors:** Nicholas Paul Holmes, Dennis Martin, William Mitchell, Zeeshan Noorani, Amber Thorne

**Affiliations:** 1 School of Psychology, University of Nottingham, Nottingham, NG7 2RD UK; 2 School of Psychology and Clinical Language Sciences, University of Reading, Reading, RG6 6AL UK

**Keywords:** Reaction time, Multisensory, Go/NoGo, Audition, Touch, Vibrotactile

## Abstract

The brain represents the space immediately surrounding the body differently to more distant parts of space. Direct evidence for this ‘peripersonal space’ representation comes from neurophysiological studies in monkeys, which show distance-dependent responses to visual stimuli in neurons with spatially coincident tactile responses. Most evidence for peripersonal space in humans is indirect: spatial- and distance-dependent modulations of reaction times and error rates in behavioural tasks. In one task often used to assess peripersonal space, sounds near the body have been argued to speed reactions to tactile stimuli. We conducted four experiments attempting to measure this distance-dependent audiotactile interaction. We found no distance-dependent enhancement of tactile processing in error rates or task performance, but found some evidence for a general speeding of reaction times by 9.5 ms when sounds were presented near the hand. A systematic review revealed an overestimation of reported effect sizes, lack of control conditions, a wide variety of methods, post hoc removal of data, and flexible methods of data analysis. After correcting for the speed of sound, removing biased or inconclusive studies, correcting for temporal expectancy, and using the trim-and-fill method to correct for publication bias, meta-analysis revealed an overall benefit of 15.2 ms when tactile stimuli are accompanied by near sounds compared to sounds further away. While this effect may be due to peripersonal space, response probability and the number of trials per condition explained significant proportions of variance in this near versus far benefit. These confounds need to be addressed, and alternative explanations ruled out by future, ideally pre-registered, studies.

**Electronic supplementary material:**

The online version of this article (10.1007/s00221-020-05771-5) contains supplementary material, which is available to authorized users.

## Introduction

The space immediately surrounding the body seems to be represented by specific multisensory neurons in parietal, premotor, and sub-cortical areas of the macaque monkey brain (Bufacchi and Iannetti 2018). Evidence for this representation of space comes primarily from animal studies; however, several lines of evidence are compatible with the existence of peripersonal space in humans (Holmes 2013). One central feature of peripersonal space is that it is multisensory: visual stimuli within a particular portion of space (a visual receptive field) are represented relative to a particular body part, for example, relative to a particular tactile receptive field on the skin (Graziano et al. 1994). Most of the evidence from animal studies comes from this visual–tactile domain.

There is very little evidence for a representation of auditory peripersonal space in monkeys. While macaque ventral intraparietal cortex has some neurons with somatosensory, visual, and auditory responses, the majority of bimodal visual–tactile responses did not use a common frame of reference (Avillac et al. 2005), and auditory stimuli have not been systematically tested. In the premotor cortex, about half of similar multisensory neurons were found to be sensitive to auditory distance (Graziano et al. 1999), responding within 50 ms of stimulus onset. Many of these cells had inhibitory responses to nearby sounds, but the source of, or effective cues for, this auditory distance sensitivity were not determined. In humans, auditory–somatosensory interactions are more often present for head-related, than hand-related regions of space (Occelli et al. 2011, however, see Serino 2019).

In part, the lack of evidence for auditory peripersonal space in macaques and humans may simply be due to a lack of appropriate studies. However, absolute perception of distance in the auditory domain is poor, biased, and ambiguous. When distance can be perceived, it is heavily dependent on the stimulus type, on the properties of the room that the listener is in, and requires sufficient time for the observer to perceive reverberation or temporal modulation of the stimulus; familiarity with the sound source is often required (Kolarik et al. 2016; Kopco et al. 2020). These properties of auditory distance perception are not helpful for a representation of peripersonal space that is hypothesised to respond quickly to unexpected stimuli, for example in defensive situations (Bufacchi and Iannetti 2018). Since little is known about any unambiguous cues to absolute auditory distance, it remains unknown whether the brain can extract the information required to represent auditory–tactile peripersonal space. The likely cues are distance-dependent changes in inter-aural level differences, frequency spectra, or direct-to-reverberant energy ratios (Kolarik et al. 2016; Kopco et al. 2020).

Despite this potential obstacle, numerous studies have investigated the effects of sounds at various distances from the body on participants’ ability to rapidly detect and discriminate tactile stimuli. Kitagawa et al. (2005) found that white noise bursts presented 20 cm away from one side of the head decreased participants’ reaction times (RT) by 5.5 ms for tactile stimuli on the same side, as compared to sounds 70 cm away. Conversely, RT was increased by 13.5 ms when presented on the opposite side (i.e. a spatial compatibility effect of 19 ms). When pure tones were presented, this distance-dependent modulation of the spatial compatibility effect was only 1 ms. This left/right spatial compatibility effect may be particularly sensitive to sound distance. As sounds approach the head from one side, the head creates an acoustic ‘shadow’—relative sound intensity and spectral frequency distribution between the ears changes. This left–right disparity increases with proximity (Kolarik et al. 2016). Distance-dependent modulations of behaviour with lateralised sounds may therefore reflect the brain's processing of sound intensity or frequency, rather than distance (Kopco et al. 2020).

Many reports have followed these left–right spatial discrimination studies, using a different experimental task. In the first such study, Serino et al. (2007) presented electrocutaneous pulses at very weak intensities (90% detection during training) on the participant’s right index finger, held near their right knee. These tactile targets were accompanied with a 150 ms burst of white noise presented near to (~ 5 cm) or far from (~ 125 cm) the hand, on the right side of space. Trials with weak target stimuli were intermixed with trials with strong, non-target stimuli, as well as with catch trials without tactile stimuli. Participants responded by saying ‘tah’ when they detected weak targets, and refrained from responding when strong or no stimuli were presented (a ‘Go/NoGo’ task). Serino and colleagues found that participants responded 36 ms earlier when the target was accompanied by a near as compared to a far sound. Using very similar methods, distance-dependent modulations of tactile RTs were also reported by Bassolino et al. (2010), Serino et al. (2011), and Cimmino et al. (2013). Using a range of different but related methods, similar effects have often been reported (Serino 2019).

The majority of evidence for audiotactile peripersonal space has come from a single laboratory. To expand the evidence base, we tested the hypothesis that sounds presented near to the hand improve the detection of tactile stimuli on the finger, designing our experiments based on the first reports in the series, by Serino et al. (2007) and Bassolino et al. (2010).

## Experiment 1

We designed an experiment as close as possible to Serino et al.’s (2007) study; the main differences were that participants were not holding or using an object (comparable to Serino et al.’s 2007 ‘before tool use, ‘passive’ and ‘handle’ conditions), and the tactile target was 125 ms white noise (comparable to Serino et al.’s, 2015 stimuli). We also counterbalanced the hand that participants used to feel the vibrations (dominant, non-dominant), and whether participants wore a blindfold before and during the experiment [Serino et al.’s (2007) participants were blindfolded]. We implemented a different thresholding procedure to set target intensity. Participants responded by saying ‘tah’ as quickly as possible to near-threshold (‘weak’) vibrotactile stimuli presented on their index finger and thumb, while attempting to ignore simultaneous sounds near to or far from their hand. The hypothesis was that reaction times would be shorter when target stimuli were accompanied by sounds near versus far from the hand.

### Methods

#### Participants

Sixteen participants consented (13 female, mean ± SD age = 21.1 ± 4.4 years, 1 left-handed, 15 right-handed by self-report). Two participants could not complete the training tasks and were removed before the experiment began. The final sample had 14 participants (11 female, 1 left-handed). Procedures were approved by the local research ethics committee (reference: 2013_138_NH), and were conducted in accordance with the Declaration of Helsinki (2008 version). Data collection occurred in 2013–2014. Sample size was based on the prior reports (*N* = 16, Serino et al. 2007; Bassolino et al. 2010). Effect sizes in these studies (Cohen’s *d* = 0.92, 0.91, respectively), implied that 16 participants provided 95% statistical power to detect the effect with  = 0.05 (92% power with 14 participants).

#### Apparatus

Vibrotactile stimuli were presented via an Oticon bone-conducting vibrator. Two Goodmans Active 46 speakers were positioned 5 cm and 105 cm away from the participant's responding hand, in approximate alignment with the participant's head, without the near speaker occluding the line of sight between the head and the far speaker. Two 10 mm-diameter red LEDs were mounted in a box, just above a 1 cm square response button. A microphone was used to record vocal responses. A National Instruments PCIe-6321 data acquisition card was used to present analogue stimuli (one tactile, two auditory) and collect responses (vocal, button press). A custom circuit was used to switch acoustic stimulation between near and far speakers. Experiments were programmed and data analysed in Matlab. All raw data, summary data, programs and analysis scripts are available at https://osf.io/73x59.

#### Design

Participants completed four blocks of 40 trials (total 160 trials). Each block comprised six conditions in a 2 × 3 design in which the variable stimulus (none, weak, strong) was crossed with the variable auditory distance (near, far). Within each block, there were 8 trials (20%) with no vibrotactile stimulus (4 with a near sound, 4 with a far sound), 16 trials with a weak target (40%), and 16 with a strong non-target (40%). Half of the vibrations were accompanied by a near, and half a far sound. Half of the participants used their non-dominant hand to feel the vibrations and their dominant to respond, and the other half used the opposite arrangement. Half of each of these sub-groups wore a mask over their eyes before and during the experiment, and the other half did not.

#### Procedure

After giving written, informed consent, participants sat facing the speakers with their hand resting on their thigh. The microphone was positioned in front of the participant’s mouth. The vibrotactile stimulator was held between the index finger and thumb throughout. The buttons were placed in their other, responding hand. The procedures were explained, then participants began the training (thresholding) session.

##### Training

Participants performed a two-interval forced-choice (2IFC) vibrotactile detection task in which a single vibration (125 ms white noise, 8 kHz sampling, 5 ms rise and fall) was presented in the middle of one of two 1 s intervals. Interval onset was signalled by a 250 ms LED flash, on the left (first) and right (second interval). Intervals were separated by 500 ms. A 2.25 s response period followed the second interval. Participants pressed the left button to indicate that the vibration was in the first, and the right to indicate the second interval. Incorrect or missing responses were followed by two 250 ms flashes of both LEDs. The next trial started after 1 s. Trials without responses were repeated. 44 trials were run. The first four trials were 'warm-up', with the target presented at high intensity (0.75, arbitrary units). In the remaining 40 trials, target intensity was adjusted using the QUEST algorithm in PsychToolBox3 (Watson and Pelli 1983, threshold = 90%, beta(slope) = 3.5, delta = 0.05, gamma = 0.5, grain = 0.005). White noise at ~ 80 dB was played throughout training to mask any sounds produced by the targets.

##### Audio-tactile task

Participants performed a Go/NoGo task in which they responded to 'weak' stimuli by saying 'tah' as quickly as possible. They were instructed to withhold responses if there was no stimulus (catch trial) or if there was a strong stimulus (non-target). The weak target intensity was set by the 2-IFC thresholding procedure to result in approximately 90% correct detection. The strong target intensity was set to be 50% higher than the weak intensity (Serino et al. 2007), and all participants could clearly perceive the (suprathreshold) strong stimulus; however, only a single trial without background noise was presented during training to check that the strong stimulus was perceptible. All trials were accompanied by a sound (125 ms white noise, 8 kHz sampling, 5 ms rise and fall, ~ 80 dB recorded at the head for all sounds). Near sounds began at vibrotactile onset. Far sounds began 2.9 ms earlier, to correct for the speed of sound. Auditory and tactile stimuli consisted of the same waveform—they were congruent—to maximise the potential for audiotactile interactions and to increase auditory localisability. Each trial began after a 1–2 s delay (pseudorandom, uniform distribution), lasted 2.6 s, and was followed by a 1 s inter-trial interval. 40 trials were performed in pseudorandom order in each of four blocks. Vibrotactile and auditory stimuli and microphone responses were recorded at 8 kHz, from 100 ms before stimulus presentation. Vocal RTs were calculated and displayed on each trial. The experimenter used this information to encourage participants to respond more quickly, but no other feedback was given.

#### Analysis

RT was defined as the first recorded sample which exceeded 10 standard deviations above baseline (the last 100 ms before stimulus presentation). During an exploratory analysis of Experiment 1, a band pass filter was applied with the high pass systematically varied between 0.1 and 2 Hz, the low pass between 50 Hz and 1 kHz, and the SD threshold between 0 and 20 SDs. The settings which gave a good trade-off between minimising false positive and false negative detections of RT were determined visually, blind to whether the data were for near or far stimulus trials. The optimal settings were 0.25–50 Hz bandpass and 10 SD criterion.

In conventional ‘signal detection’ experiments, d-prime estimates participants’ ability to distinguish between signal (target) and noise (non-targets). The criterion, C, that participants use to decide whether a particular level of sensation is a target or not can also be estimated. In Experiment 1, there were three vibrotactile conditions: none, weak, and strong. To decide whether a particular sensation was a target, participants must therefore maintain two separate response criteria—one lower criterion to distinguish no stimulus from weak targets, and a higher criterion to distinguish weak targets from strong non-targets. The experimental design used by Serino et al. (2007) and Experiment 1 here are difficult to analyse and interpret. To address this difficulty, we coded stimuli in three categories: 0 (none), 1 (weak), or 2 (strong), and responses in four: 0 (none), 1 (anticipated, RT < 150 ms), 2 (timely, 150 ms ≤ RT ≤ 2 s), and 3 (delayed, RT > 2 s). ‘Hits’ were timely responses to weak stimuli, as a proportion of all trials with weak stimuli. ‘False alarms’ were timely responses when there was no stimulus or a strong non-target stimulus, as a proportion of all trials with no target. Summary data are reported as mean ± SD, unless otherwise stated.

### Results

Performance on the main task was very poor (d-prime = -0.04 ± 0.68) and not significantly different from chance, *t*(13) = 0.22, *p* = 0.83. There were no RTs shorter than 150 ms (anticipations) and very few (2.14 ± 1.83 per participant) slow responses (Supplementary Table 1). Poor performance was due to participants incorrectly responding on 12% of trials without a stimulus, and on 63% of trials with a strong stimulus—they were not able to discriminate weak stimuli from strong stimuli. Performance did not depend on who wore a blindfold or not, or who used their dominant versus non-dominant hand (all *t*(12) < 1.85, *p* > 0.09).

**Fig. 1:**
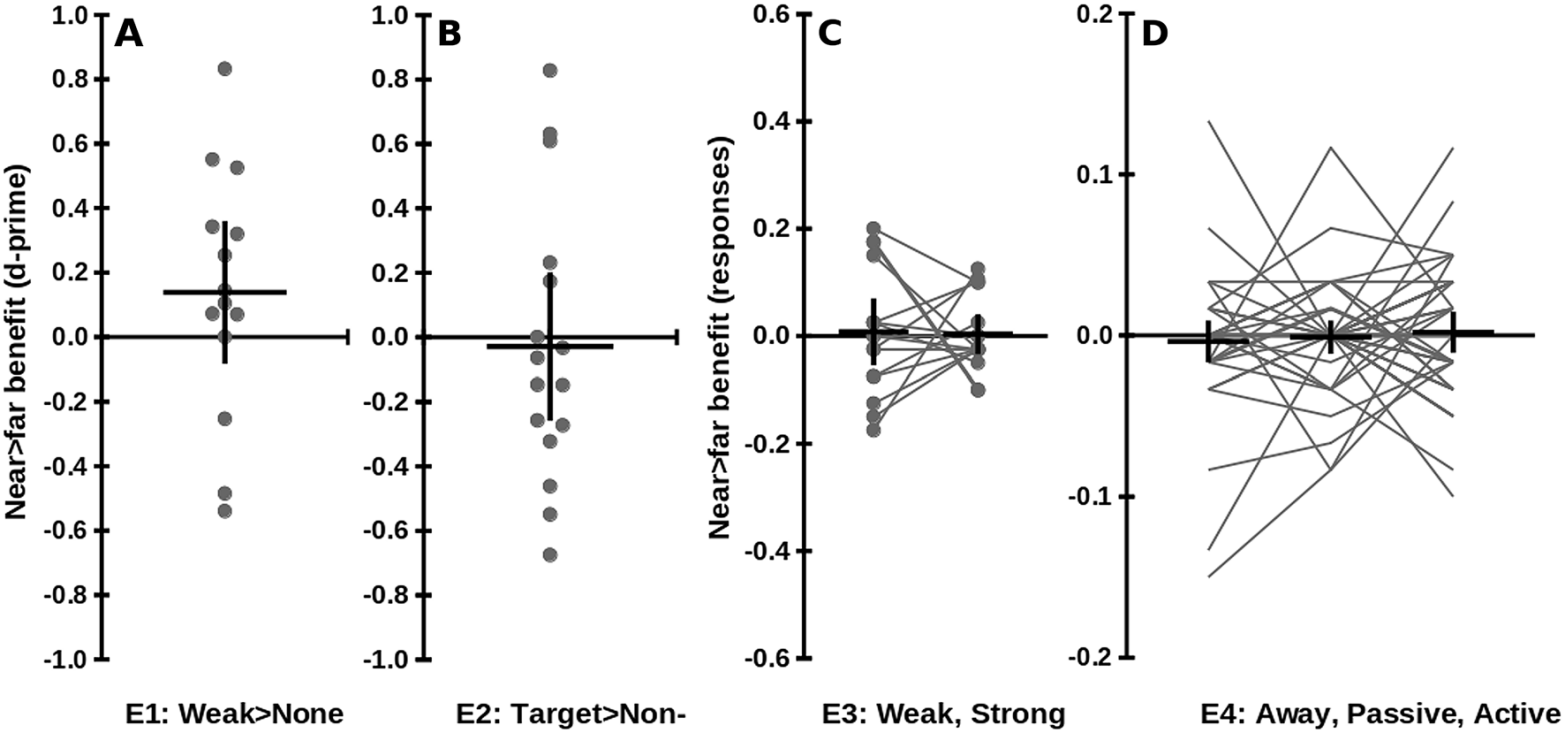
No benefit of near versus far sounds on tactile perception accuracy. Positive values show better tactile performance with near versus far sounds. Large black crosses: mean ± 95% confidence intervals, can be interpreted as a two-sided *t* test with  = 0.05. Grey circles: individual participants’ data; grey lines connect individuals. None of the differences from zero were significant. **a** Experiment 1: near > far differences in d-prime for detecting weak targets as compared to no targets and strong targets. **b** Experiment 2: near > far differences in d-prime for targets versus non-targets. **c** Experiment 3: near > far differences in proportion of responses to weak and strong targets. **d** Experiment 4: near > far differences in proportion of responses in Away, Passive, and Active conditions

**Fig. 2:**
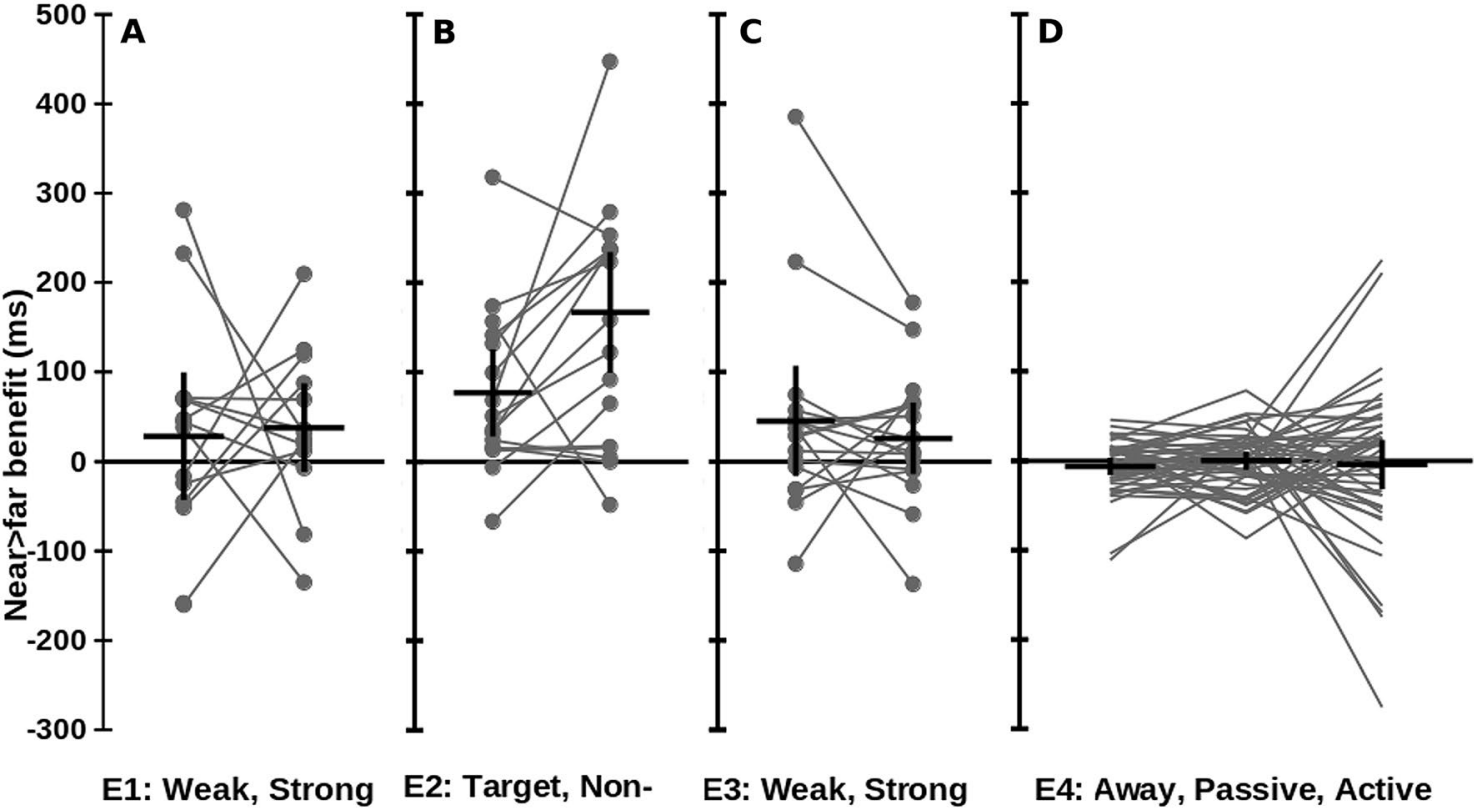
Inconsistent overall benefit of near versus far sounds on tactile reaction times (RT). Positive values show shorter tactile RT with near versus far sounds. Large black crosses: mean ± 95% confidence intervals, can be interpreted as a two-sided *t* test with  = 0.05. Grey circles: individual participants’ data; grey lines connect individuals. Differences from zero were significant only in Experiment 2. **a** Experiment 1: near > far RT differences detecting weak targets and strong non-targets (i.e. false alarms). **b** Experiment 2: near > far RT differences for targets and non-targets. **c** Experiment 3: near > far RT differences for weak and strong targets. **d** Experiment 4: near > far RT differences in Away, Passive, and Active conditions

D-prime for trials with near (0.03 ± 0.69) was similar to those with far sounds (− 0.11 ± 0.74, difference = 0.14 ± 0.38, *t*(13) = 1.35, *p* = 0.20), and neither was significantly different from zero (chance, Fig. 1a). There were no significant differences between near and far sounds for hits, *t*(13) = 1.23, *p* = 0.239; false alarms, *t*(13) = 0.988, *p* = 0.341; or criterion, *t*(13) = 0.196, *p* = 0.847 (Supplementary data). For trials with near auditory stimuli, RTs were shorter than those with a far stimulus, but this occurred regardless of whether no stimulus (near = 972 ± 340 ms, far = 985 ± 253 ms, difference in 9 participants with both responses = 123 ± 324 ms, *t*(8) = 1.12, *p* = 0.29), a weak target stimulus (near = 877 ± 194 ms, far = 905 ± 201 ms, difference = 28 ± 123 ms, *t*(13) = 0.86, *p* = 0.41), or a strong non-target stimulus was presented (near = 788 ± 195 ms, far = 826 ± 183 ms, difference = 38 ± 85 ms, *t*(13) = 1.67, *p* = 0.12, Fig. 2a). ANOVA revealed no significant main effects or interactions for RT data. A Bayesian analysis using the hypothesised effect (Serino et al. 2007; mean ± SD effect size of 35.5 ± 38.7 ms, Cohen’s *d* = 0.917, half-normal effect size) found that the data were insensitive, both for RTs to the target and non-target stimuli (Bayes factors, BF_10_ = 0.925, 2.03, respectively; Dienes 2014).

### Discussion

In line with Serino et al. (2007), participants made vocal responses on average 28 ms earlier when target vibrotactile stimuli on the hand were accompanied by sounds presented near the hand, compared to 1 m away. Responses to non-target stimuli (false alarm errors) were also earlier with near sounds, and discrimination was improved with near stimuli. However, none of these effects were statistically reliable, and discrimination remained at chance. Participants seemed to find the task too difficult. In their original report, Serino et al. (2007) trained participants to detect the weak targets to ~ 90% correct, and set the strong targets to 100% detection. No further training to discriminate between stimuli was reported. We followed these methods as closely as possible, implementing the established QUEST thresholding procedure to achieve ~ 90% threshold. What we did not anticipate was that performance during the main task would be at chance levels. Discriminating weak targets (~ 90% detection) from both no target and from strong non-targets (1.5 × stronger, 100% detection) was too difficult for our participants.

Two possibilities for this task difficulty are first that the three-way discrimination task, between no target, weak targets, and strong non-targets, was too difficult, and second that the stimulus intensity difference between weak and strong stimuli was too small for participants to discriminate reliably. We addressed these issues first, in Experiment 2, by removing the catch trials, and second, in Experiment 3, by removing the requirement to discriminate between stimuli based on intensity. The primary manipulation was always sound distance.

## Experiment 2

Experiment 2 repeated Experiment 1, but followed a more conventional Go/NoGo design. Half of the trials contained a target and required a response; half contained no target and required no response. There were no catch trials, so participants only had to maintain a single response criterion to discriminate between 'weak' and 'strong' stimuli.

### Methods

Sixteen new participants were recruited (14 female, mean ± SD age = 20.1 ± 0.9 years, 1 left-handed by self-report). The experimental design was altered by removing catch trials and increasing the number of trials in the other four conditions to 10 in each of the four blocks, giving a total of 160 trials. Half of the participants responded when they detected a weak stimulus, as in Experiment 1, while the other half responded only to strong stimuli. All other details were identical to Experiment 1.

### Results

Discrimination between target and non-target stimuli was improved relative to Experiment 1, with a mean ± SD d-prime of 0.30 ± 0.62 (between experiments, *t*(28) = 1.44, *p* = 0.162), but this was still not significantly above chance, *t*(15) = 1.96, *p* = 0.07. Blindfolded participants responded earlier (712 ± 205 ms) than non-blindfolded (916 ± 110 ms, *t*(14) = 2.48, *p* = 0.03), but d-prime scores and comparisons between dominant and non-dominant hand groups were not significant (*t*(14) = 0.99, *p* = 0.33).

For both sound distances, participants could not discriminate between targets and non-targets, with d-prime for near (0.29 ± 0.66) and far sounds (0.32 ± 0.66) not significantly different from zero or each other (difference = 0.03 ± 0.43, *t*(15) = 0.27, *p* = 0.79, Fig. 1b). There were no significant differences between near and far sounds in hits, *t*(15) = 0.132, *p* = 0.897, false alarms, *t*(15) = 0.729, *p* = 0.477, or criterion, *t*(15) = 0.589, *p* = 0.565 (Supplementary Materials). RTs to the target were significantly shorter when near sounds (780 ± 186 ms) compared to far sounds were presented (857 ± 207 ms, difference = 77 ± 91 ms, *t*(15) = 3.37, *p* = 0.004). This was also true for non-targets, with near (683 ± 153 ms) resulting in much shorter RTs than far sounds (850 ± 209 ms, difference = 167 ± 127 ms, *t*(15) = 5.26, *p* < 0.001, Fig. 2b). ANOVA on RTs revealed significant main effects of target type (*F*(1,15) = 6.78. *p* = 0.02, RTs were longer to target (819 ± 191 ms) than non-target stimuli (767 ± 172 ms)) and distance (*F*(1,15) = 29.6, *p* < 0.001, with near (732 ± 161 ms) associated with shorter RTs than far sounds (854 ± 202 ms)). A significant interaction between target and distance, *F*(1,15) = 7.72, *p* = 0.014, was explained by a larger effect of stimulus distance for non-targets (167 ± 127 ms) than for targets (77 ± 91 ms). Bayesian analyses under the same assumptions as Experiment 1 revealed strong support for a near > far benefit for RTs to target *and* non-target stimuli (BF_10_ = 97.9, 4295, respectively).

### Discussion

Experiment 2 replicated the results of Experiment 1—despite being trained to detect vibrotactile targets to ~ 90% correct performance, when accompanied by sounds near to or far from the hand, discrimination remained at chance, and participants instead seemed to respond according to the auditory distractor—responding much quicker (77 ms for targets; 167 ms for non-targets) when sounds were presented near compared to far from the hand. This was despite clear instruction to attend and respond only to target stimuli, and to ignore sounds and non-targets. It seems that participants still found the intensity discrimination task too difficult.

## Experiment 3

To further simplify the task, in Experiment 3, we removed the requirement to respond only to targets, and instead asked participants to respond to all vibrotactile stimuli, regardless of intensity. The task was therefore one-interval speeded detection.

### Methods

Sixteen new participants were recruited (12 female, mean ± SD age = 20.1 ± 0.7 years, 1 left-handed by self-report). The experimental design was altered from Experiment 2 by requiring participants to respond to every vibrotactile stimulus that they felt, both weak and strong. Participants were told that there would be a target stimulus on every trial, but were instructed to respond only when they felt it. There were 40 trials per block and four blocks (total 160 trials).

### Results

Participants responded on a mean ± SD of 76.1 ± 18.2% of trials, which was significantly lower than the expected performance for weak stimuli alone (i.e. 90%), *t*(15) = 3.04, *p* = 0.008, Supplementary Table 1. There were no substantial differences between performance in participants who were blindfolded or not, or who used their dominant hand or not (all *t*(14) < 1.72, *p* > 0.18).

Participants responded more often when strong (82.4 ± 18.4%) than when weak stimuli were presented (69.9 ± 23.2%, difference = 12.6 ± 20.7%, *t*(15) = 2.43, *p* = 0.028), but RTs were comparable (weak = 639 ± 178 ms, strong = 633 ± 165 ms, difference = 6 ± 56 ms, *t*(15) = 0.41, *p* = 0.69). None of the comparisons between responses with near and far sounds were significant for the error data (*p*s > 0.79, Fig. 1c).

RTs to weak targets were slightly shorter when a near (617 ± 184 ms) than a far sound was presented (662 ± 190 ms, difference = 45 ± 115 ms, *t*(15) = 1.57, *p* = 0.14). This trend was similar for strong targets (near = 621 ± 167 ms, far = 646 ± 171 ms, difference = 26 ± 75 ms, *t*(15) = 1.38, *p* = 0.19, Fig. 2c). Combining data from strong and weak targets did not give a significant effect of distance (difference = 36 ± 84 ms, *t*(15) = 1.69, *p* = 0.11). ANOVA revealed no significant main effects or interactions for RT data. Bayesian analyses were insensitive for RTs both to weak and strong targets (Bayes’ factors = 2.01, 1.11, respectively).

### Discussion

In Experiment 3, participants were told there was a target stimulus on every trial, but they should only respond when they felt it. Presenting a sound near compared to far from the hand did not significantly improve detection or RT. However, participants responded significantly more often on trials with strong compared to weak stimuli, suggesting that the stimuli were, as in Experiment 2, discriminable. Despite training to ~ 90% correct performance in the 2-IFC training task, participants only responded on 76% of trials. This suggests that the addition of auditory stimulation near and far from the hand impaired performance.

The 2-IFC task used in training was likely not ideal for calibrating stimulus intensity in the main experiment, in which a 1-IFC design was used. 2-IFC tasks give participants two potential stimuli, and require relative judgements, while 1-IFC tasks provide only a single potential stimulus, requiring an absolute judgement. Further, training was with continuous white noise, whereas the experiment included discrete bursts of white noise. Although other studies have not implemented this control during training, discrete sounds presented with tactile stimuli may make tactile detection worse in general, and training should include discrete sounds. In a small supplementary experiment, we tested whether participants could perform the intensity discrimination task better than chance at all. While one participant highly trained in tactile detection and discrimination tasks (the author, NPH) could perform the task, two less-trained participants found it very difficult (Supplementary Materials). This difficulty may be due to the task, the stimuli, the intensities, or a combination of factors. Future work needs to address this more thoroughly.

In Experiments 1 and 2, participants were unable to perform the task better than chance, and instead seemed to respond according to the sound location—responding sooner to trials with a near compared to a far sound location. Experiment 3 resulted in better performance on the tactile task, but no differences in RT were seen between near and far sounds. Across Experiments 1–3, the accuracy of participants’ responses did not consistently discriminate between targets and non-targets, or between weak and strong stimuli, and near sounds did not improve tactile performance (Fig. 1a–c). However, participants did seem to respond to the location of the irrelevant sounds, in general responding earlier when a near sound was presented (Fig. 2a–c).

Following these failures to find consistent improvements in tactile perception with near compared to far sounds, we ran an additional experiment aimed to test the possibility that more localisable sounds (i.e. amplitude-modulated white noise as compared to pure tones) might result in clearer effects of auditory distance on tactile perception. Unfortunately, due to a coding error, distance and auditory stimulus type were confounded, so these data were unusable (they are available at https://osf.io/73x59). One final experiment in this series was designed based on Bassolino et al. (2010).

## Experiment 4

In a final experiment, auditory–tactile interactions were investigated in the context of using a computer driving simulator. Moving a computer mouse with your hand results in a displacement of a cursor on screen. This motor–visual relationship led Bassolino et al. (2010) to test for ‘expansion of peripersonal space’ contingent on use of the mouse. In place of a mouse, we used a realistic desktop driving simulator: participants placed their hands on or near a steering wheel (*Darkfire PS3 Racing Wheel, Speedlink*) and passively watched or actively played the driving simulator game. Participants’ task was to respond by pressing a foot pedal whenever they felt a tactile stimulus on their right hand. Auditory distractors were presented near or far from the hand. Following the mixed evidence for a benefit for near stimuli in Experiments 1–3, we increased the sample size. Previous effect sizes for near > far benefits were a mean ± SD of 27.1 ± 31.2 ms (*d* = 0.876, *g* = 0.775) and 21.3 ± 22.8 ms (*d* = 0.936, *g* = 0.828) in Bassolino et al.’s (2010) Experiments 1 and 2, respectively. Using the smaller mean and the larger SD, 99% power to detect a significant effect at *p* ≤ 0.05 requires 40 participants (https://www.stat.ubc.ca/~rollin/stats/ssize/n1.html).

### Methods

#### Participants

Forty-six psychology students (39 female, mean ± SD age = 20.9 ± 3.6 years, 4 left-handed by self-report) were recruited. Two participants’ data were unusable due to equipment failures during testing (responses were not recorded). Participants were asked whether they drive.

#### Materials

The vibrotactile stimulator was attached to the participant’s right index finger or to the rear side of a computer steering wheel. Sounds were presented through a speaker positioned at 70 cm (far) from the participant’s finger and next to a computer monitor directly in front of the participant, and another 2.5 cm away (near). Foot pedals were positioned under the participant’s feet, with the ‘accelerator’ pedal to the right of the ‘brake’ pedal. A 5 min, first-person perspective video of someone playing the *Driving Simulator 2013 (*Excalibur Games*)* was shown to participants in two of the conditions.

#### Design and procedure

Participants performed three conditions in a repeated measures design, with condition order counterbalanced across participants. In the ‘Away’ condition, the target vibrotactile stimulator was attached to the participant’s right index finger and their hands rested on the table next to the steering wheel while they watched the driving simulator video. In the ‘Passive’ condition, the vibrator was attached to the right side of the steering wheel, which participants held with both hands, as if driving, but there was no relationship between the position of the steering wheel and the driving simulator video. In the ‘Active’ condition, the vibrator was on the steering wheel, and participants were playing the *Driving Simulator 2013* game. For 17 participants, there was a 5 min break between each block of the experiment, in which no stimulation was given. For the remaining 27 participants, no specific time limit was set for the breaks between blocks, and participants continued in their own time. In all conditions, participants’ hands were approximately 30 cm from their body, although this was not explicitly measured.

In each condition, participants performed two tasks: watching (or playing) the driving simulator, and responding as quickly as possible to occasional vibrotactile target stimuli presented to the right index finger. With each tactile target stimulus, an auditory distractor was presented near to or far from the participant’s hand. Both tactile targets and auditory distractors consisted of a car horn stimulus (438 ms long). Participants were instructed to press the ‘brake’ foot pedal as soon as they felt the tactile stimulus. 60 targets were presented in a single block in each of the three conditions, 30 with near and 30 with far sounds, in pseudorandomised order. RT and missed responses were dependent variables.

### Results

Participants responded on a mean ± SD of 94.4 ± 9.5% of trials, and responded late on 9.9 ± 12.2% of trials. There were no substantial differences in errors between near and far sounds (Fig. 1d), in drivers versus non-drivers, or in participants who took 5 min breaks between blocks or not (18 uncorrected comparisons, all *t*(42) < 1.93, all *p* > 0.06). ANOVA revealed no significant main effects or interactions for misses, but a significant effect of condition for late responses, *F*(2,86) = 9.33, *p* < 0.001, with more late responses in the Active (4.48 ± 8.46) compared to the Away (3.00 ± 7.86) and Passive conditions (2.52 ± 5.66).

RTs in the Active (697 ± 176 ms) were significantly longer than in the Passive (581 ± 170 ms, *t*(43) = 5.05, *p* < 0.001), and Away conditions (608 ± 197 ms, *t*(43) = 4.10, *p* < 0.001). Within each condition, there was no significant effect of sound distance (3 uncorrected comparisons, all *t*(43) < 1.29, *p* > 0.21, Fig. 2d). The effect of stimulus distance was not significantly different between the conditions (three uncorrected comparisons, all *t*(43) < 1.00, *p* > 0.33). There were no significant effects of driver status or whether participants took 5 min breaks, on the near–far differences within each condition (six uncorrected comparisons, all *t*(43) < 1.85, *p* > 0.08). ANOVA revealed only a significant main effect of condition, *F*(2,86) = 17.4, *p* < 0.001, described above. Bayesian analyses revealed, for the Away, Passive, and Active conditions, BF_10_ = 0.160, 0.087, and 0.219 respectively. The same conclusion was reached when using effect sizes from Bassolino et al. (2010) , mean ± SD effect = 27.3 ± 31.2 ms, *d* = 0.876, *g* = 0.775, BF_10_ = 0.197, 0.113, and 0.275 respectively). All showed substantial support for the null hypothesis of no difference in tactile RTs when accompanied by near versus far sounds.

### Discussion

Despite an increased sample size, over 99% power to detect the previously reported effect sizes, and a simplified task of responding to all clearly suprathreshold tactile targets, we found no evidence for or changes in distance-dependent audiotactile interactions in RT or errors. One possibility, raised by a reviewer, is that both the near (2.5 cm from the hand, ~ 30 cm from the body) and the far (70 cm from the hand, ~ 100 cm from the body) were inside the peripersonal space centred on the trunk (Galli et al. 2015; Noel et al. 2015a, b; Serino et al. 2015). However, from the ten relevant experiments reported in these three studies, the mean ± SD difference that we should expect in tactile RT between sounds near (~ 30 cm) and far (~ 100 cm) from the body was a benefit of 37 ± 33 ms (Supplementary Tabe 4). This corresponds to a very large effect size (> 1 SD) for near versus far sounds, but we found no such difference. After four experiments, given our surprising failures to find support for the large effects reported by Serino et al. (2007) and Bassolino et al. (2010), we performed a systematic review and meta-analysis of previous studies of audiotactile peripersonal space.

### Systematic review and meta-analysis of audiotactile peripersonal space

#### Method

Searching PubMed using the terms “audi* AND tact* AND (spac* OR spat*)” revealed 78 records. We searched full papers for inclusion criteria: healthy adult participants, sounds presented at two or more distances, tactile stimuli, errors and/or reaction times reported. 23 articles were deemed relevant. We extracted the experiment number, sample, task, distances, number, and type of sounds, type of tactile stimuli, type of response, body parts stimulated, biased analysis methods, the criterion for peripersonal space, number of errors, RTs for near and far stimuli, and effect sizes. If more than two auditory distances were tested, only the furthest and nearest were used. The meta-analysis aimed to provide an effect size, in ms for RT and error or d-prime for accuracy.

### Results

#### Systematic review

Across 23 articles, there were seven kinds of experiments, differing in number of auditory distances tested, kinds of auditory and tactile stimuli, body part stimulated, response required, and criteria used to test the hypothesis. Across 46 relevant experiments, there were 28 different combinations of these experimental parameters (Supplementary Tables 2 and 3). No more than four papers used the same methods, making synthesis difficult. There was little reporting of error rates or signal detection measures, despite most experiments requiring participants to respond only to targets and not non-targets—when signal detection analysis is critical. We can therefore only assess RT effects, and we note that these RT effects may have been affected or confounded by changes in response criterion or speed–accuracy trade-offs.

##### Criterion for assessing peripersonal space

Most studies used a simple effect of distance to assess peripersonal space, by comparing ‘far’ with ‘near’ sounds. However, six kinds of experiments defined peripersonal space as a difference between a particular sound distance and a tactile ‘baseline’, rather than as a difference between ‘near’ and ‘far’ sounds (Noel et al. 2015a; Pfeiffer et al. 2018 Experiment 1; Serino et al. 2015; Tonelli et al. 2019). Because these ‘baseline’ data were not acquired in balanced factorial designs including all possible time points with both tactile and audiotactile conditions, or included in the ANOVA, the reported post hoc comparisons using them are invalid and do not explain the reported effects.

This omission was fixed by Pfeiffer et al. (2018, Experiments 1 and 3), using a full factorial design. The criterion for peripersonal space was then a significant sound × distance interaction, which allowed the confounding expectancy effect to be assessed for the first time (see Kandula et al. 2017 for visual–tactile analyses). In experiments beginning with Canzoneri et al. (2012), the sounds were changing or moving, and between 1.1 and 4 s duration. The target tactile stimulus then occurred at one of several times before, during, or after the sound. As this 1–4 s passes without a target, the target then becomes increasingly likely to occur at subsequent time points (a 'hazard function'). This expectancy effect likely influenced participants’ RTs in many reports (Ardizzi and Ferri 2018; Canzoneri et al. 2012, 2013a, b; Ferri et al. 2015a, b; Galli et al. 2015; Hobeika et al. 2018; Maister et al. 2015; Noel et al. 2015a, b; Serino et al. 2015; Teneggi et al. 2013; Tonelli et al. 2019), but was controlled for only by Pfeiffer et al. (2018)—RTs were approximately 16 ms shorter for targets later as compared to earlier in the trial.

##### Control conditions

In experiments presenting one of two unchanging sounds at two distances, far sounds controlled for near sounds (Bassolino et al. 2010; Cimmino et al. 2013; Kitagawa et al. 2005; Serino et al. 2007, 2011; Tajadura-Jiménez et al. 2009; Teramoto et al. 2013). By contrast, in experiments using two speakers, the relative intensity of each speaker was adjusted to produce a ‘looming’ (increasing intensity of near relative to far), or ‘receding’ sound (increasing far relative to near, Ardizzi and Ferri 2018; Canzoneri et al. 2012, 2013a, b; Ferri et al. 2015a; Maister et al. 2015; Serino et al. 2015; Teneggi et al. 2013). Initially, receding and looming conditions were analysed according to the time that the target was presented, and both sounds were argued to enhance tactile processing, although receding sounds had smaller effects (Canzoneri et al. 2012). In two later reports, looming and receding sounds were analysed by the perceived distance of the sounds, pooled and interpreted together (Canzoneri et al. 2013a, b). In other experiments, the sounds were analysed separately, the authors arguing that only looming sounds affected audiotactile interactions (Noel et al. 2015a; Serino et al. 2015). In yet other experiments, receding sounds were replaced with constant sounds (Ardizzi and Ferri 2018; Ferri et al. 2015a, b) sometimes excluded from analysis (Ferri et al. 2015b), or no control sound was used (Ferri et al. 2015b; Galli et al. 2015; Maister et al. 2015; Noel et al. 2015b; Serino et al. 2015; Teneggi et al. 2013).

##### Types of stimuli and responses

Sounds have included changes in intensity played over headphones (Ferri et al. 2015a, b), which cannot provide distance information (Kolarik et al. 2016), and are thus irrelevant to peripersonal space. Other studies used headphone-presented sounds filtered using generic head-related transfer functions to create more realistic spatial sound (Hobeika et al. 2018; Pfeiffer et al. 2018), or arrays of two (Serino et al. 2007), four (Galli et al. 2015), seven (Tonelli et al. 2019), or eight (Noel et al. 2015a, b) speakers. The sounds varied between constant white noise (Serino et al. 2007), white noise presented from different speakers sequentially (Galli et al. 2015), changing amplitude pink noise (Canzoneri et al. 2012), different kinds of noise (Ferri et al. 2015b), and water bubbling (Hobeika et al. 2018). In some reports, the near sound onset was adjusted to account for the speed of sound (343 m/s or 2.9 ms/m), sometimes rounded up to 5 ms (Bassolino et al. 2010; Serino et al. 2007, 2011). Other papers did not report this correction; these later studies overestimated RTs for targets with far sounds, and the near > far sound effect size.

Tactile stimuli have been pulses of near- or above-threshold electrical stimulation (Serino et al. 2007), vibrations (Galli et al. 2015), or air puffs (Teramoto et al. 2013); presented to the finger (Serino et al. 2007), arm or hand (Canzoneri et al. 2012), cheek (Teneggi et al. 2013), ear (Kitagawa et al. 2005; Tajadura-Jiménez et al. 2009), chest or back (Galli et al. 2015), neck (Tonelli et al. 2019), or head (Serino et al. 2015; Pfeiffer et al. 2018).

Participants responded vocally (Serino et al. 2007), manually (Ferri et al. 2015a, b), or pedally (Kitagawa et al. 2005; Tajadura-Jiménez et al. 2009; Teramoto et al. 2013). RTs were analysed in raw form, after log-transforming and rescaling (Ferri et al. 2015a, b), after re-scaling alone (Ardizzi and Ferri 2018), using ANOVA (most reports), or fitting a sinusoidal or linear model to the data (Ardizzi and Ferri 2018; Canzoneri et al. 2012; Ferri et al. 2015a, b; Teneggi et al. 2013).

##### Task

Most experiments used a Go/NoGo design: on some trials participants responded to a tactile target, and on others they withheld responses. The percentage of Go trials varied between 40 and 94%. Some experiments required responses on every trial (Kitagawa et al. 2005; Maister et al. 2015; Tajadura-Jiménez et al. 2009; Teramoto et al. 2013). We found a significant negative correlation between the proportion of Go trials, and the overall benefit for near versus far sounds, across the reviewed literature, *r*(71) = − 0.295, *p* = 0.011 (Supplementary Fig. 1). About 9% of the variance in the benefits of near sounds may therefore be related to response preparation effects. For each increase in response probability of 0.1, the near > far effect is reduced by about 5 ms. This effect may be substantially different for visual–tactile versions of this task (Kandula et al. 2017). Furthermore, following a reviewer’s question, we also found a similar negative correlation between the number of trials per condition in each experiment and the overall benefit for near versus far sounds, *r*(71) =  − 0.442, *p* < 0.001, 20% of variance explained (Supplementary Fig. 2). This suggests that shorter experiments with fewer trials per condition are significantly associated with larger apparent effects of audiotactile peripersonal space, perhaps due to publication bias. Together, the proportion of trials with responses and the total number of trials collected per condition explained 24% of the variance in the near > far benefit. Dedicated studies are required to examine these effects in more detail.

#### Bias

RT outliers (more than 2 or 2.5 SD from the mean) were often removed, though little detail was provided. Fixed outlier cutoffs can bias data (Van Selst and Jolicoeur 1994). In some studies, analyses fitting RT data by a model were restricted to participants with 'acceptable' fits (Ardizzi and Ferri 2018; Ferri et al. 2015a, b; Teneggi et al. 2013). Since the hypothesis predicted that participants would show significant fits, removing poorly fitting participants biases the dataset and overestimates effect size. Ferri et al. (2015b) removed three participants with poor fit from each of two experiments, removed the control condition from analysis in experiment 1, and removed one of the four stimulus conditions in experiment 2. Ardizzi and Ferri (2018) excluded participants with poor sigmoidal fits, then concluded that sigmoidal fits were significantly better than linear fits. This is circular (Holmes 2007). Finally, in an experiment testing whether learning to 'echo-locate' influences peripersonal space, Tonelli et al. (2019) excluded participants based on worse performance in the last 30 compared to the first 10 trials of a two-alternative forced-choice task. Although described as “inability to complete training” (p. 856), six of the included participants' performance was not significantly better than chance (i.e. < 19/30, binomial test).

#### Meta-analysis

Given the heterogeneity in tasks, stimuli, responses, controls, and analysis, we only asked the simplest possible question: how much shorter are RTs when accompanied by near compared to far sounds? We implemented adjustments in a stepwise manner to attempt to ‘correct’ or counteract the biases and heterogeneity in this literature. First, we pooled data across conditions within the same groups of participants, to protect against dependent effects (Hedges et al. 2010). Second, we corrected RTs for the speed of sound. Third, we removed studies which biased their data by post hoc selection (see above), and Experiments 1–2 reported here, in which participants could not perform the task. Fourth, we corrected for the estimated 'expectancy effect' (Pfeiffer et al. 2018) by subtracting (looming) or adding (receding) 16 ms to the reported near > far benefit. Fifth, we re-assessed and corrected for the relationship between response likelihood and near > far benefit, assuming that all tasks required responses on every trial. Sixth, we re-assessed and corrected for the relationship between the number of trials per condition and the near > far benefit, regressing out this relationship around the mean number of trials per study. Seventh, at each of the above stages, we performed a trim-and-fill analysis to assess any resulting funnel plot asymmetry and adjust for potential publication bias (Duval and Tweedie 2000). The meta-analytic effect size after each of these steps, based only on those studies in which an effect size and its standard error could be estimated, is shown in Table 1.

**Table 1:** Effect of different stepwise corrections on overall meta-analytic effect size

Correction applied	Prior to trim and fill					Publication bias correction (trim and fill)				
	k studies	Mean ± SE effect (ms), Cohen’s *d*^a^	CI (ms)	*Z*	*p*	m studies	Mean ± SE effect (ms), Cohen’s *d*^a^	CI (ms)	*Z*	*p*
None	33	34.7 ± 4.6 0.484	{25.8,43.8}	7.57	< .001	38	31.5 ± 4.6 0.439	{22.6,40.4}	6.93	< 0.001
Independent groups	28	37.7 ± 4.5 0.526	{28.8,46.5}	8.35	< .001	33	33.1 ± 4.7 0.462	{24.0,42.3}	7.10	< 0.001
Speed of sound	28	36.4 ± 4.4 0.508	{28.8,46.5}	8.33	< .001	32	33.2 ± 4.4 0.463	{24.6,42.0}	7.52	< 0.001
Selection bias and experiment failure	23	29.8 ± 3.0 0.416	{23.9,35.6}	9.97	< .001	34	20.7 ± 4.2 0.289	{12.5,28.9}	4.96	< .001
Temporal expectancy	23	25.7 ± 3.7 0.358	{18.5,32.9}	7.00	< .001	35	15.2 ± 3.8 0.212	{7.7,22.8}	3.96	< 0.001
Proportion responses	23	16.7 ± 3.2 0.233	{10.3,23.0}	5.15	< .001	33	9.9 ± 3.5 0.138	{3.0,16.8}	2.80	0.005
Trials per condition	23	17.9 ± 3.4 0.250	{11.2,24.6}	5.21	< .001	38	5.9** ± **3.7 0.082	{-1.4,13.1}	1.58	0.115

*ms* milliseconds, *k* number of data points, *SE* standard error of effect size, *CI* 95% confidence interval, *Z* meta-analytic *Z* score across the included studies. The first five columns of data show the meta-analysis results before correcting for publication bias. The final five columns show the same results after the trim-and-fill method was applied. *m* number of studies, including studies imputed following the trim-and-fill method

^a^Cohen’s *d* is calculated using the pooled within-study, between-participants SD of 71.7 ms

Many studies provided only point (rather than spread) estimates of the near versus far benefit. Across all studies, the corrected near > far benefit varied from − 42 ms (performance worse with near than far, Teramoto et al. 2013) to + 81 ms (Teneggi et al. 2013, Experiment 3). Across our Experiments 3–4, where participants could perform the task above chance, random effects meta-analysis (JASP 0.9.2) revealed a non-significant overall RT benefit of near over far stimuli of 9.5 ms, with 95% confidence intervals {− 26.1, 45.2} ms, *Z* = 0.52, *p* = 0.60.

**Fig. 3:**
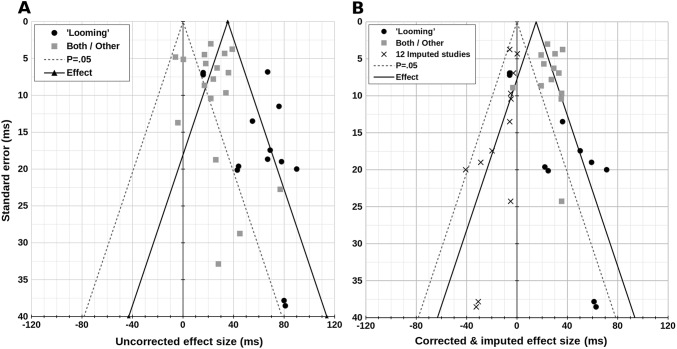
Contour-enhanced funnel plots of the uncorrected (**a**), and the corrected and imputed (**b**) reaction time (RT) benefit of near versus far sounds on tactile detection. *X* axes: near > far benefit in ms. *Y* axes: standard error in ms. Positive X values show shorter RTs with near versus far sounds. Black circles: ‘looming’ sounds (approaching or increasing intensity). Mid-grey squares: either both ‘looming’ and ‘receding’ sounds were not reported separately, or the sounds did not change. Broken grey lines: contour for effects that would pass the standard 5% alpha criterion—studies inside the triangle have *p* > 0.05, studied outside *p* < 0.05. Solid black lines: overall meta-analytic mean effect size. **a** Shows all raw, uncorrected effect sizes. **b** RTs were pooled within groups and corrected for the speed of sound (2.9 ms/m). Biased or failed studies were removed, and studies with ‘looming’ stimuli were corrected for the temporal expectancy effect. Publication bias was assessed and corrected using the trim-and-fill method (Duval and Tweedie 2000). These effect sizes are not corrected for the proportion of Go trials, or the number of trials tested per condition. Incorporating both these (arguable) corrections to the dataset would reduce the publication bias-corrected meta-analytic effect size (Table 1)

Across all studies, after correcting for the speed of sound, removing failed and biased studies, and correcting for the expectancy effect, the meta-analytic effect size was 25.7 ms {18.5, 32.9} ms, *Z* = 7.00, *p* < 0.001. The resulting funnel plot appeared asymmetric (Fig. 3a), suggesting publication bias. The trim-and-fill algorithm converged after eight iterations, and suggested there are 12 ‘suppressed’ or missing effect sizes, along with 23 published effect sizes. Filling-in these missing effects resulted in a meta-analytic mean near > far benefit of 15.2 ms {7.7, 22.8} ms, *Z* = 3.96, *p* < 0.001, Fig. 3b. This effect size does not take into account the proportion of Go trials or the number of trials per condition, which differ substantially across studies and explain a significant portion of variance in the near > far benefit (Supplementary Figs. 1 and 2). Including both of these additional adjustments reduced the meta-analytic mean effect size to 5.9 ms {− 1.4, 13.1} ms, *Z* = 1.58, *p* = 0.115.

## General discussion

Sounds presented near versus far from the hands did not significantly improve tactile RT or performance in Experiments 1, 3, or 4. Experiment 2 showed a strong and significant decrease in RT with near stimuli; however, this was regardless of stimulus type, and participants performed their main task at chance. Bayesian analyses revealed that Experiments 1 and 3 were insensitive, 2 was supportive of a near over far benefit, and 4 provided strong evidence for no effect. Meta-analysis across Experiments 3 and 4 revealed a 9.5 ms benefit in RTs for near versus far sounds. This benefit was not statistically significant and was smaller than the overall literature’s mean effect size after publication bias was assessed (15.2 ms). Given that the between-participant pooled SD for the effect was 71.7 ms (Cohen’s *d* = 0.212), a typical study in this literature (mean sample size = 16), would have 21% power to detect this effect. A replication study would require 138 participants to achieve 80% power to detect a one-tailed benefit of near > far sounds on tactile RT at the conventional 5% false-positive rate. If the proportion of Go trials and overall number of trials were also taken into account, more participants may be required. We first discuss potential limitations of our work, before speculating about alternative mechanisms for generating a near > far benefit in RT.

### Limitations

Experiments 1–3 were designed to reproduce the results of Serino et al. (2007). As such, the intensity of the weak target stimuli was set such that participants could detect around 90% of the weak targets. No detail of the procedure used to set target intensity was provided by Serino et al. (2007), although some later reports do provide more detail. In the pre-test training session, we used a two-interval forced-choice thresholding procedure (QUEST) to set the weak target intensity at ~ 90% correct. During the main experiment, however, we used a one-interval design, and performance was lower—this is the expected outcome when comparing one- versus two-interval designs. We also only presented continuous auditory masking noise during pre-test training, but discrete bursts of noise during the main experiments. Although we were able to find only one other report where the authors may have used auditory masking noise “throughout the experiment” (Teramoto et al. 2013, p 429), discrete bursts of noise may interfere more with tactile detection than continuous noise. While possible, this is not the conclusion that typical studies of audiotactile peripersonal space made. In much of this literature, it is claimed that auditory stimuli enhance tactile perception (i.e. decrease RT, although few effects on accuracy have been reported). If repeating Experiments 1–3, we would ensure that 90% of weak stimuli could be detected under audiotactile conditions identical to the main experiment, and that weak and strong stimuli could be more clearly discriminated under the main experimental conditions.

In each experiment, we collected at least 30 trials per condition per participant (32 in E1, 40 in E2-E3, 30 in E4). This is similar to the report of Serino et al.' (2007, 30 trials) and more than most of the reports that we included in our meta-analysis (e.g. there were 8 trials per condition in Canzoneri et al. 2012, 2013a, b, and Teneggi et al. 2013; see Supplementary Table 2). It is unlikely that insufficient data collection accounts for our failures to replicate audiotactile peripersonal space effects. We also found no effect of block order (Supplementary Fig. 3), suggesting that performance did not change with increasing numbers of trials).

Our Experiment 1 closely, but not exactly followed Serino et al. (2007) (and Experiment 4 was further still from Bassolino et al.’s 2010). One could always claim that this was why we could not reproduce the reported effects. However, the systematically reviewed literature used 28 different combinations of the most-relevant experimental manipulations (Supplementary Tables 2 and 3). Within this literature, many experimental manipulations and situations were claimed to modify or be sensitive to audiotactile peripersonal space, including blindness (Serino et al. 2007), computer mouse use (Bassolino et al. 2010), surgical arm elongation (Cimmino et al. 2013), rake use (Canzoneri et al. 2013a), amputation and prosthesis implantation (Canzoneri et al. 2013b), sitting opposite people (Teneggi et al. 2013), playing cooperative games (Teneggi et al. 2013), interpersonal multisensory stimulation (Maister et al. 2015), negative sounds (Ferri et al. 2015a, b), wheelchair use (Galli et al. 2015), ‘full body’ visuotactile illusions (Noel et al. 2015b), body posture (Serino et al. 2015), handedness (Hobeika et al. 2018), body rotations (Pfeiffer et al. 2018), and learning to ‘echolocate’ (Tonelli et al. 2019). It is perhaps unlikely, given the large heterogeneity in this literature revealed here, both that all of these reported effects are reliable and that the particular experimental decisions we made were insufficient to reproduce these effects. We now discuss two alternative explanations for a benefit of near over far sounds in tactile RT: peripersonal space and alerting.

### Peripersonal space

There may be an RT advantage for sounds near the body because they activate a representation of audiotactile peripersonal space (Serino 2019). This would fit with a large body of work on visual–tactile peripersonal space (Holmes 2013). However, there is little evidence of audiotactile peripersonal space in monkeys, and only a little is known about how the brain might compute auditory distance (Kolarik et al. 2016; Kopco et al. 2020). Furthermore, studies using non-spatial sounds (Ferri et al. 2015a, b) found similar effects to those using virtual or free-field sounds, suggesting that auditory distance is irrelevant to generating these RT benefits of ‘near’ stimuli. If an audiotactile peripersonal space exists, can quickly extract and represent auditory distance, and has body part-centred receptive fields, why would this create RT advantages in tactile detection? Since the reported near over far benefit in RT occurs with both near-threshold (e.g. Serino et al. 2007) and supra-threshold tactile stimuli (e.g. Serino et al. 2015), it is unlikely due to the multisensory facilitation of near-threshold somatosensory inputs. Perhaps, near sounds activate more neurons in the peripersonal population, and this somehow leads to earlier motor responses? Or perhaps they activate neurons more likely to evoke or facilitate motor responses? The mechanism by which audiotactile peripersonal space decreases tactile RT needs to be further specified and tested. Some computational modelling work on visuotactile (Kandula et al. 2017; Magosso et al. 2010) and audiotactile (Noel et al. 2018) peripersonal space has been done; however, Noel et al.’s (2018) audiotactile model assumed, as an input, the existence of a highly topographic map of auditory distance. This is computationally the most difficult part, and evidence for the existence of such a detailed representation in the brain is scarce (Kolarik et al. 2016). Auditory representations of distance based only on the direct-to-reverberant energy ratio seem possible in humans (Kopco et al. 2020), but distance discrimination is difficult (i.e. the best forced-choice performance for frontal stimuli was around 80% correct), requires training, familiarity with the stimulus, and sufficient time to perceive room reverberations (likely > 500 ms). It is unknown whether putative mechanisms of peripersonal space have access to representations of auditory distance that could facilitate rapid tactile RTs, which were often reported to be less than 500 ms.

### Alerting

One alternative explanation for earlier responses with near than far sounds is that nearby or looming sounds may be more alerting or aversive (Neuhoff 2016). Nearby sounds may cause more startling responses than far sounds, and this startle may lead to shorter RT (Marinovic and Tresilian 2016). This does not, however, solve the problem. Why are nearby or looming sounds more alerting? This could be due to auditory features that vary with distance, but are these differences present in the stimulus (i.e. absolute cues to distance) or learned over time (relative cues)? This alerting effect explanation is supported by our unexpected observation that the largest RT benefits of nearby sounds were seen in experiments with the smallest proportions of Go trials, while little RT benefit was seen in simple RT tasks in which a response was required every trial, as explained next.

The alerting effect of nearby sounds may be particularly strong for responses that are given less frequently, or when responses are not being prepared. The Go/No task is complex: different response preparation and inhibition processes depend on the proportion of responses required, the time between trials, and the instructions to participants (Ficarella and Battelli 2019). Decreasing the probability of Go trials from 75 to 25%, for example, leads to RT increases of 140 ms (Low and Miller 1999). The range of Go trial proportions in the Go/NoGo studies reviewed here was 40–94%. In our Experiments 1 (40% Go) and 2 (50% Go), raw Go/NoGo RTs were 223 ms longer than in the simple RT tasks (Experiments 3–4). We speculate that similar general RT increases occurred in Go/NoGo tasks used in studies of audiotactile peripersonal space. With this general increase in Go/NoGo RT, there is then more potential for decreases in RT caused by the presentation of relatively more startling (nearby or looming) sounds.

## Conclusion

We found no evidence for a benefit of near versus far sounds in our experiments. Our largest experiment found strong evidence for no effect, despite having over 99% power to detect it. Our systematic review and meta-analysis suggested that the most important methodological factors for future researchers to address are: (1) the proportion of trials requiring a response (Supplementary Fig. 1), (2) the number of trials per condition (Supplementary Fig. 2), (3) control conditions for temporal expectancy (Pfeiffer et al., 2018; Kandula et al. 2017), (4) control of all non-spatial aspects of auditory stimulation, (5) measurement and control of speed–accuracy trade-offs, and (6) reporting bias, such that studies with low precision and small or negative effects have not been published. These issues should be addressed in dedicated, ideally preregistered, studies which systematically control and manipulate these factors. Until we know how these potentially interacting factors contribute to the auditory distance-related modulation of tactile reaction times, we find it difficult to make specific or strong conclusions about the magnitude or mechanisms of any representation of audiotactile peripersonal space in humans.

## Electronic supplementary material

Below is the link to the electronic supplementary material.

Supplementary file1 (ODS 162 kb)

Supplementary file2 (ODS 125 kb)

Supplementary file3 (DOC 671 kb)

Supplementary file4 (ODS 317 kb)
