# Supplementary material for: Do sounds near the hand facilitate tactile reaction times? Four experiments and a meta-analysis provide mixed support and suggest a small effect size
Source: Exp Brain Res. 2020 Mar 19;238(4):995–1009. doi: 10.1007/s00221-020-05771-5 (PMC7181441; doi:10.1007/s00221-020-05771-5)
Supplement: Supplementary file 3 — Supplementary file3 (DOC 671 kb) [file 221_2020_5771_MOESM3_ESM.doc]

***Supplementary Table 1: Mean±SD percentage response types and reaction times in Experiments 1-3***

|  |  | **Mean±SD responses (percentage, RT)** | | |
| --- | --- | --- | --- | --- |
| **Expt.** | **Stimulus** | **None** | **On time** | **Delayed** |
| **1** | **None** | 87.7±7.9% | 11.2±7.2%; 975±266ms | 1.1±1.6%; 2252±171ms |
|  | **Weak** | 55.0±17.8% | 43.2±18.1%; 890±183ms | 1.8±2.3%; 2275±130ms |
|  | **Strong** | 37.4±22.4% | 61.6±22.1%; 808±183ms | 1.0±1.3%; 2136±99ms |
| **2** | **Target** | 45.7±21.2% | 53.8±21.4%; 814±190ms | 0.5±1.3%; 2273±161ms |
|  | **Non-target** | 56.3±21.0% | 42.7±21.7%; 768±179ms | 0.9±1.7%; 2300±110ms |
| **3** | **Weak** | 29.6±23.2% | 70.0±23.2%; 635±176ms | 0.6±0.6%; 2292±194ms |
|  | **Strong** | 17.0±17.8% | 82.3±18.6%; 634±165ms | 0.5±0.1%; 2249±31ms |

Expt.: Experiment; SD: standard deviation; RT: reaction time

***Supplementary Table 2: Systematic review of the audio-tactile peripersonal space literature***

| **Citation** | **E** | **N** | **Stimuli (% trials)** | **Sound during training** | **Trials per condition** | **Body part** | **Task**  **(% trials)** | **Response** | **Outliers**  **(% cut)** | **PPS criterion** | **Miss %** | **FA %** | **Reaction times (ms)** | **Distance effect (Far-Near)** | **SD ratio**  **(Between / Within)** | **Effect size** |
| --- | --- | --- | --- | --- | --- | --- | --- | --- | --- | --- | --- | --- | --- | --- | --- | --- |
|  |  |  |  |  |  |  |  |  |  |  | **Mean**  **(SD)** | **Mean**  **(SD)** | **Mean**  **(SD)** | **Mean**  **(SD) ms** |  | **Cohen’s d** |
|  |  |  | **TWO SPEAKERS, STATIC WHITE NOISE, ELECTRICAL STIMULUS ON R INDEX, VOCAL RESPONSE** |  |  |  |  |  |  |  |  |  |  |  |  |  |
| Serino et al. 2007 | 1 | 16 | T+: Elec weak 0.9(?) (40)  T-: Elec strong 1.0(?) (40)  AN: WN .15s 70dB 5cm (50)  AF: WN .15s 70dB 125cm -5ms (50) | ? | 30NT+  30FT+  30NT-  30FT-  15N  15F | R index | Go (40)  NoGo (60) | ‘Tah’ | >2SD (<4%) | Distance effect  (Far-Near) | ~12.5() | <1() | N:663()  F:697() | 34(38.7) | - | 0.88 |
| Bassolino et al 2010 | 1 | 16 | T+: Elec weak 0.9(1I-2AFC) (57)  T-: Elec strong 1.0(1I-2AFC) (34)  AN: WN 0.1s 70dB 15cm (50)  AF: WN 0.1s 70dB 72cm -5ms (50) | ? | 25NT+  25FT+  15NT-  15FT-  4N  4F | R index | Go (57)  NoGo (43) | ‘Tah’ | >2SD (3.8%) | Distance effect  (Far-Near) | N:8(12)  F:5(8) | N:1(4)  F:4(4) | N:750(140)  F:774(148) | 24(31.2) | 4.61 | 0.77 |
| Bassolino et al 2010 | 2 | 16 | T+: Elec weak 0.9(1I-2AFC) (56)  T-: Elec strong 1.0(1I-2AFC) (37)  AN: WN 0.1s 70dB 15cm (35)  AF: WN 0.1s 70dB 72cm -5ms (35) | ? | 25NT+  25FT+  15NT-  15FT-  4N  4F  20T+  20T- | R index | Go (56)  NoGo (44) | ‘Tah’ | >2SD (2%) | Distance effect  (Far-Near) | N:2(4)  F:4(8)  X:0(0) | N:1(4)  F:1(4)  X:0(0) | N:582(136)  F:600(152)  X:() | 18(22.8) | 6.31 | 0.79 |
| Serino et al. 2011 | 1A | 12 | T+/-: Elec 0.1ms weak 0.9(1I-2AFC) (47)  T+/-: Elec 0.1ms strong 1.0(1I-2AFC) (47)  AN: WN 0.1s 70dB 5cm (34)  AF: WN 0.1s 70dB 100cm -5ms (34) | ? | 20WN  20WF  20SN  20SF  4N  4F  20W  20S | R index | Go (47)  NoGo (53) | ‘Tah’ | >2SD (4%) | Distance effect  (Far-Near) | N:2()  F:3()  X:2() | N:<1()  F:<1()  X:<1() | N:524(69)  F:541(83)  X:569(87) | 17(~30) | 2.66 | ~0.57 |
| Serino et al. 2011 | 1B | 18 | T+: Elec 0.1ms weak 0.9 1I-2AFC (43)  T-: Elec 0.1ms strong 1.0 1I-2AFC (43)  AN: WN 0.1s 70dB 5cm (50)  AF: WN 0.1s 70dB 100cm -5ms (50) | ? | 30NT+  30FT+  30NT-  30FT-  10N  10F | R index | Go (43)  NoGo (57) | ‘Tah’ | >2SD (3.9%) | Distance effect  (Far-Near) | 2() | 1() | N:597(136)  F:624(132) | 27(26.7) | 5.02 | 1.01 |
| Serino et al. 2011 | 2 | 10 | T+: Elec 0.1ms weak 0.9 1I-2AFC (43)  T-: Elec 0.1ms strong 1.0 1I-2AFC (43)  AN: WN 0.1s 70dB 5cm (50)  AF: WN 0.1s 70dB 100cm -5ms (50) | ? | 30NT+  30FT+  30NT-  30FT-  10N  10F | R index | Go (43)  NoGo (57) | ‘Tah’ | >2SD (2.7%) | Distance effect  (Far-Near) | 4() | <1() | N:583(101)  F:600(108) | 17(14.2) | 7.36 | 1.20 |
| Cimmino et al. 2013 | 4 | 7 | T+: Elec 0.1ms weak 0.9(?) (50)  T-: Elec 0.1ms strong 1.0(?) (33)  AN: WN 0.15s 70dB 5cm (50)  AF: WN 0.15s 70dB 100cm -5ms (50) | ? | 60NT+  60FT+  40NT-  40FT-  20N  20F | R index | Go (50)  NoGo (50) | ‘Tah’ | >2SD (0.63%) | Distance effect  (Far-Near) | 1.62() | 0.87() | N:584(101)  F:606(106) | 22(8) | 12.9 | 2.75 |
|  |  |  | **TWO SPEAKERS, DYNAMIC PINK NOISE, ELECTRICAL STIMULUS ON R INDEX, HAND, OR ARM, VOCAL RESPONSE** |  |  |  |  |  |  |  |  |  |  |  |  |  |
| Canzoneri et al. 2012 | 1 | 17 | T+: Elec 0.1ms 1.0(1I-2AFC), -0.7:-3.6s (60)  AN: PN 3s 55-70dB 5cm (50)  AF: PN 3s 55-70dB 100cm (50) | ? | IN:  8D0  8D1  8D2  8D3  8D4  8D5  8D6  38 catch  (same OUT) | R index | Go (60)  NoGo (40) | ‘Tah’ | >2SD (1.6%) | Distance effect (IN/OUT coded by time)  (Residual and slope of sigmoid fit) | 1.75() | 0.25() | IN  N:398(62)  F:478(74)  X: 466(82)  OUT  N:432(66)  F:454(62)  X: 477(74) | 80(~156)  22(~43) | ~0.47  ~4.70 | ~0.51  ~0.51 |
| Canzoneri et al. 2013a | 1A | 12 | T+: Elec 0.1ms 1.0(?), +0.3:+2.7s (60)  AN: PN 3s 55-70dB 5cm (50)  AF: PN 3s 55-70dB 100cm (50) | ? | IN:  D1?  D2?  D3?  D4?  D5?  (same OUT?) | R index | Go (60)  NoGo (40) | ‘Tah’ | >2SD (1.5%) | Distance effect IN & OUT | 2.88() | 0.06() | IN/OUT  N:444(104)  F:477(104) | 33(~15) | ~6.93 | ~2.16 |
| Canzoneri et al. 2013a | 3 | 12 | T+: Elec 0.1ms 1.0(?), +0.3:+2.7s (60)  AN: PN 3s 55-70dB 5cm (50)  AF: PN 3s 55-70dB 100cm (50) | ? | IN:  D1?  D2?  D3?  D4?  D5?  (same OUT?) | R index | Go (60)  NoGo (40) | ‘Tah’ | >2SD  (?%) | Distance effect IN & OUT | 1.57() | 0.76() | IN/OUT  N:390(87)  F:429(90) | 39(~13) | ~6.81 | ~2.91 |
| Canzoneri et al. 2013b | 2 | 20 | T+: Elec 0.1ms 1.0(?), +0.3:+2.7s (50)  AN: PN 3s 55-70dB 5cm (50)  AF: PN 3s 55-70dB 100cm (50) | ? | IN:  81  8D2  8D3  8D4  8D5  (same OUT) | R arm | Go (50)  NoGo (50) | ‘Tah’ | >2SD (0.81%) | Distance effect IN & OUT | 2.87() | 0.86() | IN/OUT  N:404(51)  F:440(51) | 36(~31) | ~1.65 | ~1.15 |
| Canzoneri et al. 2013b | 3 | 12 | T+: Elec 0.1ms 1.0(?), +0.3:+2.7s (50)  AN: PN 3s 55-70dB 5cm (50)  AF: PN 3s 55-70dB 100cm (50) | ? | IN:  81  8D2  8D3  8D4  8D5  (same OUT) | R arm  R  hand | Go (50)  NoGo (50) | ‘Tah’ | >2SD (0.54%) | Distance effect IN & OUT | 1.89() | 0.45() | IN/OUT  N:~347(104)  F:~382(77) | ~35() | - | - |
|  |  |  | **TWO SPEAKERS, DYNAMIC PINK NOISE, ELECTRICAL STIMULUS ON R CHEEK, VOCAL RESPONSE** |  |  |  |  |  |  |  |  |  |  |  |  |  |
| Teneggi et al. 2013 | 1 | 18 | T+: Elec 0.1ms 1.0(1I-2AFC), +0.3:+2.7s (77)  AN: PN 3s 55-70dB 5cm (50)  AF: PN 3s 55-70dB 100cm (50) | ? | IN  8D1  8D2  8D3  8D4  8D5  12 catch  (same OUT) | R cheek | Go (77)  NoGo (23) | ‘Tah’ | -  -2 bad fit | Distance effect IN ONLY  Sigmoid fit PSE | 2.5(0.2) | 0.4(0) | IN  N:415(107)  F:484(93)  OUT  N:447(106)  F:443(119)  **PSE**  **A:1384()**  **B:1566()** | 69(74)  -4()  **182(455)** | ~1.35  -  **-** | ~0.93  -  **0.40** |
| Teneggi et al. 2013 | 2A | 16 | T+: Elec 0.1ms 1.0(1I-2AFC), +0.3:+2.7s (77)  AN: PN 3s 55-70dB 5cm (50)  AF: PN 3s 55-70dB 100cm (50) | ? | IN  8D1  8D2  8D3  8D4  8D5  12 catch  (same OUT) | R cheek | Go (77)  NoGo (23) | ‘Tah’ | -  -2 bad fit | Distance effect IN ONLY  Sigmoid fit PSE | 3.75(3) | 0.5(2.1) | IN  N:429(66)  F:484(74)  OUT  N:447(76)  F:459(64)  **PSE**  **Pre:1467()**  **Post:1443()** | 55(54)  8()  **24()** | 1.48  -  **-** | 1.02  -  **0.09** |
| Teneggi et al. 2013 | 2B | 16 | T+: Elec 0.1ms 1.0(1I-2AFC), +0.3:+2.7s (77)  AN: PN 3s 55-70dB 5cm (50)  AF: PN 3s 55-70dB 100cm (50) | ? | IN  8D1  8D2  8D3  8D4  8D5  12 catch  (same OUT) | R cheek | Go (77)  NoGo (23) | ‘Tah’ | -  - | Distance effect IN ONLY  Sigmoid fit PSE | 3.59(3.3) | 0.8(1.7) | IN  N:449(60)  F:526(110)  OUT  N:486(68)  F:488(56)  **PSE**  **Pre:()**  **Post:()** | 78(76)  2()  **-** | 1.12  -  **-** | 1.03  -  **-** |
| Teneggi et al. 2013 | 3 | 20 | T+: Elec 0.1ms 1.0(1I-2AFC), +0.25:+3.7s (77)  AN: PN 4s 55-70dB 5cm (50)  AF: PN 4s 55-70dB 100cm (50) | ? | IN  8D-2  8D-1  8D1  8D2  8D3  8D4  8D5  12 catch  (no OUT) | R cheek | Go (77)  NoGo (23) | ‘Tah’ | -  - | Distance effect IN ONLY  Sigmoid fit PSE | 4.0(3.4) | 0.6(0) | IN  N:~436(103)  F:~536(119)  **PSE**  **Pre:1911()**  **Post:1731()** | ~100()  **180()** | -  **-** | -  **0.47** |
|  |  |  | **TWO SPEAKERS, DYNAMIC PINK NOISE, ELECTRICAL STIMULUS ON R HAND, L FINGER RESPONSE** |  |  |  |  |  |  |  |  |  |  |  |  |  |
| Maister et al. 2015 | 1 | 16 | T+: Elec 0.2ms 1.4ST(1I-2AFC), -0.7:-3.6s (100)  AN: PN 3s 55-70dB 5cm (50)  AF: PN 3s 55-70dB 100cm (50) | ? | Pre, Synch  10D1  10D2  10D3  10D4  10D5 | R hand | Go (100) | L index | >2SD (5%) | Distance effect | 9() | () | IN  N:361(44)  F:451(56) | 90(<80) | >0.62 | >1.12 |
| Ferri et al. 2015a | 1A | 38 | T+: Elec 0.1ms 1.0(1I-2AFC), +0.3:+2.7s (60)  AN: PN 3.1s 55-70dB 5cm (50)  AF: PN 3.1s 55-70dB 100cm (50)  A0: PN 3.1s 62.5dB 5/100cm (100) | ? | 16D0  16D1  16D2  16D3  16D4  16D5  16D6  16D7  16D8  16D9  16D10  32 catch  (same per noise) | R index | Go (60)  NoGo (40) | L index | >2SD  (L:2.0%)  (F:2.1%)  **-2: head movement**  **-8 bad fit**  **(sigmoid p<.05)**  sigmoid<linear before exclusions | Distance effect (IN coded by time), log-transformed & normalised RT  **Sigmoid fit (2 parameters) PSE** | N/FL:2.8()  0N/F:2.1() | () | IN  N:302(52)  F:369(55)  Flat  0N:334(36)  0F:344(56)  **1494(411)** | 67(112)  10()  - | 0.48  - | 0.60  - |
| Ferri et al. 2015a | 2 | 20 | T+: Elec 0.1ms 1.0, +0.3:+2.7s (60)  AN: PN 3.1s 55-70dB 5cm (50)  AF: PN 3.1s 55-70dB 100cm (50)  A0: PN 3.1s 62.5dB 5/100cm (100) | ? | 12D0  12D1  12D2  12D3  12D4  12D5  12D6  12D7  12D8  12D9  12D10  40 catch  (same per noise) | R index | Go (60)  NoGo (40) | L index | -4 bad fit  (sigmoid p<.05) | Sigmoid fit (2 parameters) PSE |  |  | 1598  1561 |  |  |  |
| Ferri et al. 2015b | 3A | 20 | T+: Elec 0.1ms 1.0(1I-2AFC), +0.3:+2.7s (60)  AN: PN 3.1s 55-70dB 5cm (50)  AF: PN 3.1s 55-70dB 100cm (50)  A0: PN 3.1s 62.5dB 5/100cm (100)  Auditory-only catch trials (40) | ? | 18D1  18D2  18D3  18D4  18D5  12 catch  (per sound) | R index | Go (60)  NoGo (40) | L index | -2 bad fit  (sigmoid p<.05) | Sigmoid fit (2 parameters) PSE |  |  | 1467 |  |  |  |
| Ferri et al. 2015b | 3B | 20 | T+: Elec 0.1ms 1.0(1I-2AFC), +0.3:+2.7s (60)  AN: PN 3.1s 55-70dB 5cm (50)  AF: PN 3.1s 55-70dB 100cm (50)  A0: PN 3.1s 62.5dB 5/100cm (100)  Left-index catch trials (40) | ? | 18D1  18D2  18D3  18D4  18D5  12 catch  (per sound) | R index | Go (60)  NoGo (40) | L index | -2 bad fit  (sigmoid p<.05) | Sigmoid fit (2 parameters) PSE |  |  | 1578 |  |  |  |
| Ardizzi & Ferri 2018 | 1 | 41 | T+: Elec 0.1ms 1.0(?), +0.3:+2.7s (60)  AN: PN 3s 55-70dB 5cm (50)  AF: PN 3s 55-70dB 100cm (50)  A0: PN 3s 62.5dB 5/100cm (100) | ? | 18D1  18D2  18D3  18D4  18D5  12 catch  (per sound) | R index | Go (60)  NoGo (40) | L index | **-5 bad fit (r^2<0.7)**  **(based on sigmoid, then show sigmoid>linear fit)** | Distance effect (IN coded by time), normalised RT  **Sigmoid fit (2 parameters) PSE** | L-N/F:()  0-N/F:() | () | N:309(55)  F:376(64)  0N:()  0F:()  **1356(437)** | 67(41)  ()  - | 1.46  - | 1.66  - |
|  |  |  | **TWO SPEAKERS, DYNAMIC PINK NOISE, VIBROTACTILE STIMULUS ON HAND OR CHEST, HAND RESPONSE** |  |  |  |  |  |  |  |  |  |  |  |  |  |
| Serino et al. 2015 | 1 | 15 | T+: Vib 0.1s 1.0, 150Hz, 113mm2 ()  AN: PN 4s 55-70dB 5cm (50)  AF: PN 4s 55-70dB 100cm (50) | ? | IN  16D1  16D2  16D3  16D4  16D5  16D6  32 base  16 catch  (same OUT) | Chest | Go (93)  NoGo (7) | hand | - | **Comparison with fastest baseline** | IN:  2.6(5.6)  OUT:  2.1(5.2) | IN:  1.9(11.6)  OUT:  1.5(10.8) | I*N*  *N:-47(50)*  *F:16(77)*  *OUT*  *N:-13(74)*  *F:-26(54)* | 63()  -13() | -  - | -  - |
| Serino et al. 2015 | 3 | 16 | T+: Vib 0.1s 1.0, 150Hz, 113mm2 ()  AN: PN 4s 55-70dB 5cm (50)  AF: PN 4s 55-70dB 100cm (50) | ? | IN  16D1  16D2  16D3  16D4  16D5  16 base  8 catch  (same OUT) | Hand | Go (92)  NoGo (8) | hand | - | **Comparison with fastest baseline** | IN?  2.5(9.2)  OUT?  1.8(6.0) | IN?  2.8(8.0)  OUT?  1.9(7.2) | IN  *N:-48(40)*  *F:10(92)*  *OUT*  *N:-30(32)*  *F:-10(36)* | 58()  20() | -  - | -  - |
| Serino et al. 2015 | 4 | 18 | T+: Vib 0.1s 1.0, 150Hz, 113mm2 ()  AN: PN 4s 55-70dB 5cm (50)  AF: PN 4s 55-70dB 100cm (50) | ? | IN, hand  16D1  16D2  16D3  16D4  16D5  16D6  16 base  8 catch  (same OUT, same hand) | Hand & chest | Go (93)  NoGo (7) | hand | - | **Comparison with fastest baseline** | N:0.8(5.1)  F:0.7(7.6) | N:1.5(3.8)  F:0.9(4.2) | IN  *N:-55(47)*  *F:3(68)*  *OUT*  *N:-24(68)*  *F:-20(81)* | 58()  4() | -  - | -  - |
| Serino et al. 2015 | 5 | 19 | T+: Vib 0.1s 1.0, 150Hz, 113mm2 ()  AN: PN 4s 55-70dB 5cm (50)  AF: PN 4s 55-70dB 100cm (50) | ? | 24N1  24N2  24N3  24N4  24N5  24N6  48 base  24 catch  (same Far) | Hand | Go (89)  NoGo (11) | hand | - | **Comparison with fastest baseline** | Face:  1.3(4.8)  Trunk:  1.3(3.9) | Face:  0.8(5.2)  Trunk:  1.2(6.5) | IN  *N:-41(35)*  *F:19(48)* | 60() | - | - |
|  |  |  | **FOUR SPEAKERS, MOVING WHITE NOISE, VIBROTACTILE STIMULUS ON CHEST OR BACK, HAND RESPONSE** |  |  |  |  |  |  |  |  |  |  |  |  |  |
| Galli et al. 2015 | 1 | 12 | T+: Vib 0.1s 1.0(?), 150Hz, 113mm2 (83)  A1:4 WN .75m/s 2m, 0.38:2.28s (100) | ? | FRONT  12D1  12D2  12D3  12D4  12D5  12D6  24 baseline  12 catch  (same BACK) | Chest  Back | Go (89)  NoGo (11) | R index | <0ms  >1000ms  >2SD (1.5%) | Distance effect | ~3() | ~3() | IN  N:305(49)  F:321(49)  X:() | 16(<25) | >1.93 | >0.64 |
| Galli et al. 2015 | 2 | 12 | T+: Vib 0.1s 1.0(?), 150Hz, 113mm2 (83)  A1:4 WN .75m/s 2m, 0.38:2.28s (100) | ? | FRONT  12D1  12D2  12D3  12D4  12D5  12D6  24 baseline  12 catch  (same BACK) | Chest  Back | Go (89)  NoGo (11) | R index | <0ms  >1000ms  >2SD (1.5%) | Distance effect | ~3() | ~3() | IN  N:288(21)  F:304(33)  X:() | 16(<24) | >1.35 | >0.64 |
| Galli et al. 2015 | 3 | 12 | T+: Vib 0.1s 1.0(?), 150Hz, 113mm2 (83)  A1:4 WN .75m/s 2m, 0.38:2.28s (100) | ? | FRONT  12D1  12D2  12D3  12D4  12D5  12D6  24 baseline  12 catch  (same BACK) | Chest  Back | Go (89)  NoGo (11) | R index | <0ms  >1000ms  >2SD (1.5%) | Distance effect | ~3() | ~3() | IN  N:284(55)  F:327(55)  X:() | 44(<68) | >0.81 | >0.64 |
| Noel et al. 2015b | 1 | 19 | T+: Vib 0.1s 1.0(?), 150Hz, 113mm2 (83)  A1:4 WN .75m/s 1m, 0.38:2.28s (100) | ? | Synch  12D1  12D2  12D3  12D4  12D5  12D6  24 base  12 catch  same for asynch | Chest | Go (89)  NoGo (11) | R hand | >2.5SD  (<3%) | Distance effect | () | 2.3(15) | IN  N:317(61)  F:398(61) | 81(168) | >0.36 | >0.48 |
| Noel et al. 2015b | 2 | 15 | T+: Vib 0.1s 1.0(?), 150Hz, 113mm2 (83)  A1:4 WN .75m/s 1m, 0.19:1.14s (100) | ? | Synch  12D1  12D2  12D3  12D4  12D5  12D6  24 base  12 catch  same for asynch | Back | Go (89)  NoGo (11) | R hand | >2.5SD  (<2%) | Distance effect | () | 5.1(7.9) | IN  N:344(39)  F:387(81) | 43(78) | >1.05 | >0.55 |
|  |  |  | **SEVEN SPEAKERS, MOVING WHITE NOISE, VIBROTACTILE STIMULUS ON NECK, VOCAL RESPONSE** |  |  |  |  |  |  |  |  |  |  |  |  |  |
| Tonelli et al. 2019 | 1A | 16 | T+: Vib 20ms 1.0(?), 18mm2 (80)  A1:7 WN 0.34m/s 0.17-1.19m, -0.5:3.5s (80) | ? | 12D1  12D2  12D3  12D4  12D5  12D6  12D7  28 base  28 catch | L neck | Go (80)  NoGo (20) | ‘Tah’ | >2SD (2.6(0.5)%)  -2 training | **Comparison with fastest baseline** | - | - | IN  N:-29(49)  F:10(45) | 39() | - | - |
| Tonelli et al. 2019 | 1B | 14 | T+: Vib 20ms 1.0(?), 18mm2 (80)  A1:7 WN 0.34m/s 0.17-1.19m, -0.5:3.5s (80) | ? | 12D1  12D2  12D3  12D4  12D5  12D6  12D7  28 base  28 catch | L neck | Go (80)  NoGo (20) | ‘Tah’ | >2SD (2.6(0.5)%) | **Comparison with fastest baseline** | - | - | IN  N:-33(56)  F:1(34) | 34() | - | - |
| Tonelli et al. 2019 | 1C | 14 | T+: Vib 20ms 1.0(?), 18mm2 (80)  A1:7 WN 0.34m/s 0.17-1.19m, -0.5:3.5s (80) | ? | 12D1  12D2  12D3  12D4  12D5  12D6  12D7  28 base  28 catch | L neck | Go (80)  NoGo (20) | ‘Tah’ | >2SD (2.6(0.5)%) | **Comparison with fastest baseline** | - | - | IN  N:-43(34)  F:4(45) | 47() | - | - |
|  |  |  | **EIGHT SPEAKERS, MOVING WHITE NOISE, VIBROTACTILE STIMULUS ON CHEST, BACK, OR HEAD, HAND RESPONSE** |  |  |  |  |  |  |  |  |  |  |  |  |  |
| Noel et al. 2015a | 1 | 18 | T+: Vib 0.1s 1.0(?), 150Hz, 113mm2 (88)  A1:8 WN 50dB 0.75m/s 2m, 0.38:2.28s (75) | ? | 12D1  12D2  12D3  12D4  12D5  12D6  24 base  12 catch | Chest | Go (88)  NoGo (12) | R hand | - | **Comparison with fastest baseline** | 0(0) | 2(4.7) | *IN*  *N:-27(29)*  *F:+18(24)*  *OUT*  *N:-23(****123****)*  *F:-25(****102****)* | 45()  -2() | -  - | -  - |
| Noel et al. 2015a | 2 | 18 | T+: Vib 0.1s 1.0(?), 150Hz, 113mm2 (88)  A1:8 WN 50dB 0.75m/s 2m, 0.38:2.28s (75) | ? | 12D1  12D2  12D3  12D4  12D5  12D6  24 base  12 catch | Chest | Go (88)  NoGo (12) | R hand | - | **Comparison with fastest baseline** | 0(0) | 0.9(0.7) | I*N*  *N:-33(23)*  *F:8(25)*  *OUT*  *N:-24(****89****)*  *F:-23(****115****)* | 41()  1() | -  - | -  - |
| Serino et al. 2015 | 2 | 16 | T+: Vib 0.1s 1.0(?), 150Hz, 113mm2 ()  A1:8 WN 50dB 0.75m/s 2m, 0.38:2.28s (75) | ? | IN  12D1  12D2  12D3  12D4  12D5  12D6  12D7  12D8  12D9  12D10  12D11  12D12  12D13  18 base  12 catch  (same OUT) | Chest  Back | Go (94)  NoGo (6) | hand | - | **Comparison with fastest baseline** | IN:  1.6(6.0) | IN:  0.5(5.2) | IN  Chest  *N:-52(32)*  *F:-5(48)*  *Back*  *N:-41(40)*  *F:-12(52)* | 47()  29() | -  - | -  - |
| Serino et al. 2015 | 6 | 15? | T+: Vib 0.1s 1.0(?), 150Hz, 113mm2 ()  A1:8 WN 50dB 0.75m/s 2m, 0.38:2.28s (75) | ? | IN  16D1  16D2  16D3  16D4  16D5  16D6  16D7  32 base  16 catch  (same OUT) | Head  Chest | Go (90)  NoGo (10) | hand | - | **Comparison with fastest baseline** | Head:  1.9(7.1)  Chest:  1.5(7.2) | Head:  0.9(5.4)  Chest:  0.9(4.9) | *IN*  *Head-head*  *N:-67(46)*  *F:-27(43)*  *Head-chest*  *N:-24(62)*  *F:-26(50)*  *Chest-chest*  *N:-49(27)*  *F:-18(31)*  *Chest-head*  *N:-31(50)*  *F:-10(43)* | *40()*  *-2()*  *31()*  *21()* | *-*  *-*  *-*  *-* | *-*  *-*  *-*  *-* |
|  |  |  | **TWO SPEAKERS, STATIC WHITE NOISE, AIR PUFF STIMULUS ON HAND, FOOT RESPONSE** |  |  |  |  |  |  |  |  |  |  |  |  |  |
| Teramoto et al. 2013 | 2 | 18 | T+: Air puff, 50ms 1.0(?)  AN: WN 45º 90dB L/R behind head 20cm  AF: WN 45º 90dB L/R by knees | Y | 20NC  20NI  20FC  20FI  (per each of 4 body parts) | L/R  palm / dorsum  of hand | Go(100)  L/R discrim | L/R foot pedal | 2% errors | (raw RTs not analysed) | - | - | **Same side**  Hand near head  N:349(122)  F:346(115)  Hand near knee  N:352(125)  F:370(187)  **Opposite sides**  Hand near head  N:416(132)  F:376(131)  Hand near knee  N:410(155)  F:408(195) | -3()  18()  -40()  -2() | -  -  -  - | -  -  -  - |
|  |  |  | **TWO SPEAKERS, STATIC WHITE NOISE, ELECTRICAL STIMULUS ON EAR, FOOT RESPONSE** |  |  |  |  |  |  |  |  |  |  |  |  |  |
| Kitagawa et al. 2005 | 2 | 10 | T+: Elec 0.1s .95(1I-2AFC) (100)  AN: WN/Tone 0.15s 70dB 20cm (50)  AF: WN/Tone 0.15s 70dB 70cm (50) | ? | 50N  50F  per sound type | Ear | Go (100) | Foot | - | Distance | 2% | - | White noise  Same  N:344()  F:351()  Opposite  N:400()  F:385()  Tone  Same  N:361()  F:337()  Opposite  N:392()  F:368() | 7()  -15()  -24()  -24() | -  -  -  - | -  -  -  - |
| Tajadura-Jimenez et al. 2009 | 1 | 15 | T+: Elec 0.05s 1(1I-2AFC) (75)  AN+: WN 0.05s 50dB 20cm (37.5)  AF+: WN 0.05s 50dB 70cm (37.5) | ? | 40NT+  40FT+  40NT-  40FT-  40N  40F  80T | Ear | Go (100) | Hand | - | Distance | 2% | - | Same side  N:302()  F:312()  Opposite side  N:313()  F:317() | 10()  4() | -  - | -  - |
| Tajadura-Jimenez et al. 2009 | 2 | 12 | T+: Elec 0.05s 1(1I-2AFC) (75)  AN+: WN 0.05s 50dB 20cm (37.5)  AF+: WN 0.05s 50dB 70cm (37.5) | ? | 40NT+  40FT+  40NT-  40FT-  40N  40F  80T | Hand | Go (100) | Foot | - | Distance | 3% | - | Hand near  Same side  N:226()  F:224()  Opposite side  N:223()  F:225()  Hand far  Same side  N:227()  F:224()  Opposite side  N:226()  F:225() | -2()  2()  -3()  -1 | -  -  -  - | -  -  -  - |
|  |  |  | **HEADPHONES, DYNAMIC COMPLEX SOUNDS, VIBRATION ON HAND, HAND RESPONSE** |  |  |  |  |  |  |  |  |  |  |  |  |  |
| Hobeika et al. 2018 | 1 | 49 | T: Vib, 20ms 250Hz (91.7)  A: 3s, water, HRTF ±60deg, -0.65:3.65s, 20-135cm, 38cm/s | ? | 10D1  10D2  10D3  10D4  10D5  10D6  10D7  10D8  10D9  20 base  20 catch  per hemi-space | Nondom  index | Go (91.7)  NoGo (8.3) | Dom index | >2SD (4.5%)  -7 for handedness  -exclude T1 (370ms) because sig diff from T2 (357ms) | Effect of distance | - | - | IN  N:300(39)  F:370(49)  T-only  Before:  A0:369(32)  After  A0:332(31) | 70() | - | - |
|  |  |  | **HEADPHONES, DYNAMIC PINK OR OTHER NOISE, VIBRATION ON HAND, HAND RESPONSE** |  |  |  |  |  |  |  |  |  |  |  |  |  |
| Ferri et al. 2015a | 1B | 38 | In MRI scanner  T-: Elec 0.1ms 1.0(1I-2AFC), +0.3:+2.7s (40)  Right-index (catch) trials (60)  AI: PN 3.1s 55-70dB (50)  A0: PN 3.1s 62.5dB (50) | ? | 18D1  18D2  18D3  18D4  18D5  12 catch | L index | Go (40)  NoGo (60) | L middle | -2: head movement  -8 bad fit  **(sigmoid p<.05)** | Distance effect (IN coded by time), log-transformed & normalised RT | () | () | IN  N:388(125)  F:464(126)  0N:()  0F:() | 76(69) | 1.82 | 1.11 |
| Ferri et al. 2015b | 1,2 | 17 | T-: Elec 0.1ms 1.0(1I-2AFC), -0.5:+3.0s (85)  AI: CN 3s 55-70dB (50)  A0: CN 3s 62.5dB (50) | ? | 18D1  18D2  18D3  18D4  18D5  12 catch | L index | Go (85)  NoGo (15) | R middle | -3 bad fit | Distance effect (IN coded by time) | () | () | IN  N:296()  F:388()  0N:()  0F:() | 92() | - | - |
| Ferri et al. 2015b | 2 | 22 | T-: Elec 0.1ms 1.0(1I-2AFC), -0.5:+3.0s (77)  AI: CN 3s 55-70dB (50)  A0: CN 3s 62.5dB (50) | ? | 18D1  18D2  18D3  18D4  18D5  12 catch | L index | Go (77)  NoGo (23) | L middle | -3 bad fit | Distance effect (IN coded by time) | () | () | IN  N:320()  F:388()  0N:()  0F:() | 60() | - |  |
|  |  |  | **HEADPHONES, DYNAMIC PINK NOISE, HRTF, VIBRATION ON HEAD, HAND RESPONSE** |  |  |  |  |  |  |  |  |  |  |  |  |  |
| Pfeiffer et al. 2018 | 1 | 16 | T: Vib, 100ms 150Hz (91.7)  A: 4s, PN, HRTF ±90deg, 0.6:3.5s, 0-100cm, 50cm/s (69.6) | ? | 20D1  20D2  20D3  20D4  20D5  20D6  20D7  42 catch | Forehead | Go (91.3)  NoGo (8.7) | R index | >2.5SD  (<10%) | Distance x sound interaction; Differences from baseline | <5% | <5% | IN  Left  N:283(~50)  F:325(~50)  Right  N:286(~50)  F:330(~50)  Baseline  N:301(~50)  F:315(~50) | 42()  44()  14() | -  -  - | -  -  - |
| Pfeiffer et al. 2018 | 3 | 14 | T: Vib, 100ms 150Hz (91.7)  A: 4s, PN, HRTF ±90deg, 0.6:3.5s, 0-100cm, 50cm/s (91.7?) | ? | 20D1  20D2  20D3  20D4  20D5  20D6  20D7  42 catch | Forehead | Go (91.3)  NoGo (8.7) | R index | >2.5SD  (<10%) | Distance x sound congruency interaction  (no baseline) | <5% | <5% | IN  Left-cong  N:342(~62)  F:398(~62)  Left-incong  N:338(~62)  F:384(~62)  Right-cong  N:335(~62)  F:400(~62)  Right-incong  N:333(~62)  F:393(~62) | 56()  46()  65()  60() | -  -  -  - | -  -  -  - |

***Supplementary table 3: Previous audio-tactile experimental design and stimuli, ordered by frequency of method***

| **Study** | **PPS criterion** | **Speakers** | **Response** | **Tactile** | **Body part** | **Sounds** | **Trials/condition** | **Notes** |
| --- | --- | --- | --- | --- | --- | --- | --- | --- |
| Serino et al. 2007 | Distance | 2 | Vocal | Electrical | Finger | Constant WN | 30 | Near delayed |
| Bassolino et al 2010 | Distance | 2 | Vocal | Electrical | Finger | Constant WN | 25 | Near delayed |
| Serino et al. 2011 | Distance | 2 | Vocal | Electrical | Finger | Constant WN | 20 | Near delayed |
| Cimmino et al. 2013 | Distance | 2 | Vocal | Electrical | Finger | Constant WN | 60 |  |
| Canzoneri et al. 2012 | Distance | 2 | Vocal | Electrical | Finger | Changing PN | 8 | IN & OUT (similar) |
| Canzoneri et al. 2013a | Distance | 2 | Vocal | Electrical | Finger | Changing PN | 8? | IN & OUT (similar) |
| Canzoneri et al. 2013b | Distance | 2 | Vocal | Electrical | Arm/hand | Changing PN | 8 | IN & OUT (similar) |
| Teneggi et al. 2013 | Distance | 2 | Vocal | Electrical | Cheek | Changing PN | 8 | IN only |
| Ferri et al. 2015a E1A, E2 | Distance; Sigmoid fit | 2 | Hand | Electrical | Finger | Changing PN | 16, 12 | IN only; Log- & normalised RTs |
| Ardizzi & Ferri 2018 | Distance; Sigmoid fit | 2 | Hand | Electrical | Finger | Changing PN | 18 | IN only; Normalised RTs |
| Maister et al. 2015 | Distance | 2 | Hand | Electrical | Hand | Changing PN | 10 | IN only; No catch trials |
| Tajadura-Jimenez et al. 2009 (E1) | Distance | 2 | Hand | Electrical | Ear | Constant WN | 40 | No distance effect |
| Kitagawa et al. 2005 | Distance | 2 | Foot | Electrical | Ear | Constant WN | 50 | No distance effect / Near worse |
| Tajadura-Jimenez et al. 2009 (E2) | Distance | 2 | Foot | Electrical | Hand | Constant WN | 40 | No distance effect |
| Teramoto et al. 2013 | Distance | 2 | Foot | Air puff | Hand | Constant WN | 20 | No distance effect |
| Galli et al. 2015 | Distance | 4 | Hand | Vibration | Chest/back | Moving WN | 12 | IN only |
| Noel et al. 2015b | Distance | 4 | Hand | Vibration | Chest/back | Moving WN | 12 | IN only |
| Ferri et al. 2015a | Distance | Headphones | Hand | Electrical | Finger | Changing PN | 18 | IN only; Log- & normalised RTs; MRI |
| Ferri et al. 2015b (E1) | Distance; Sigmoid fit | Headphones | Hand | Electrical | Finger | Changing noises | 18 | IN only (control sounds not reported) |
| Ferri et al. 2015b (E2) | Distance; Sigmoid fit | Headphones | Hand | Electrical | Finger | Changing noises | 18 | IN only |
| Hobeika et al. 2018 | Distance | Headphones | Hand | Vibration | Finger | Water; HRTF | 10 | IN only |
| Serino et al. 2015 (E1,3,4,5) | Baseline | 2 | Hand | Vibration | Hand/chest | Changing PN | 16, 16, 16, 24 | IN & OUT (different) |
| Tonelli et al. 2019 | Baseline | 7 | Vocal | Vibration | Neck | Moving WN | 12 | IN only |
| Noel et al. 2015a | Baseline | 8 | Hand | Vibration | Chest | Moving WN | 12 | IN & OUT (different) |
| Serino et al. 2015 (E2) | Baseline | 8 | Hand | Vibration | Chest/back | Moving WN | 12 | IN only |
| Serino et al. 2015 (E6) | Baseline | 8 | Hand | Vibration | Chest/head | Moving WN | 16 | IN only |
| Pfeiffer et al. 2018 (E1) | Distance X Sound interaction; Baseline | Headphones | Hand | Vibration | Head | Changing PN; HRTF | 20 |  |
| Pfeiffer et al. 2018 (E3) | Distance X Sound interaction | Headphones | Hand | Vibration | Head | Changing PN; HRTF | 20 | No baseline |

WN: White noise; PN: Pink noise; HRTF: Head related transfer function; IN: Sounds increasing intensity, and/or moving towards participant; OUT: Sounds decreasing intensity and/or moving away from participant; RTs: reaction times; MRI: Experiment done during magnetic resonance imaging; E: Experiment number

***Supplementary Figure 1****. The proportion of trials on which a response was required (‘Go’ trials, x-axis) correlated negatively with the corrected near over far benefit in ms. Datapoints show individual studies, experiments, groups, or conditions. The best-fit line, y=-49.2x+70.8ms, explained a significant proportion of variance, r(71)=-0.295, p=.011, r^2=.087. The mean effect when response probability=1 is 21.6ms.*

***Supplementary Figure 2****. The number of trials per condition in each experiment correlated negatively with the corrected near over far benefit in ms. Data-points show individual studies, experiments, groups, or conditions. The best-fit line, y=-1.05x+52.6ms, explained a siginficant portion of variance, r(71)=-.442, p<.001, r^2=.195.*

*Together with the proportion of trials on which a response was required (Supplementary Figure 1), these two variables accounted for 24% of the variance in the Near>Far effect size, F(2,70)=10.8, p<.001.*


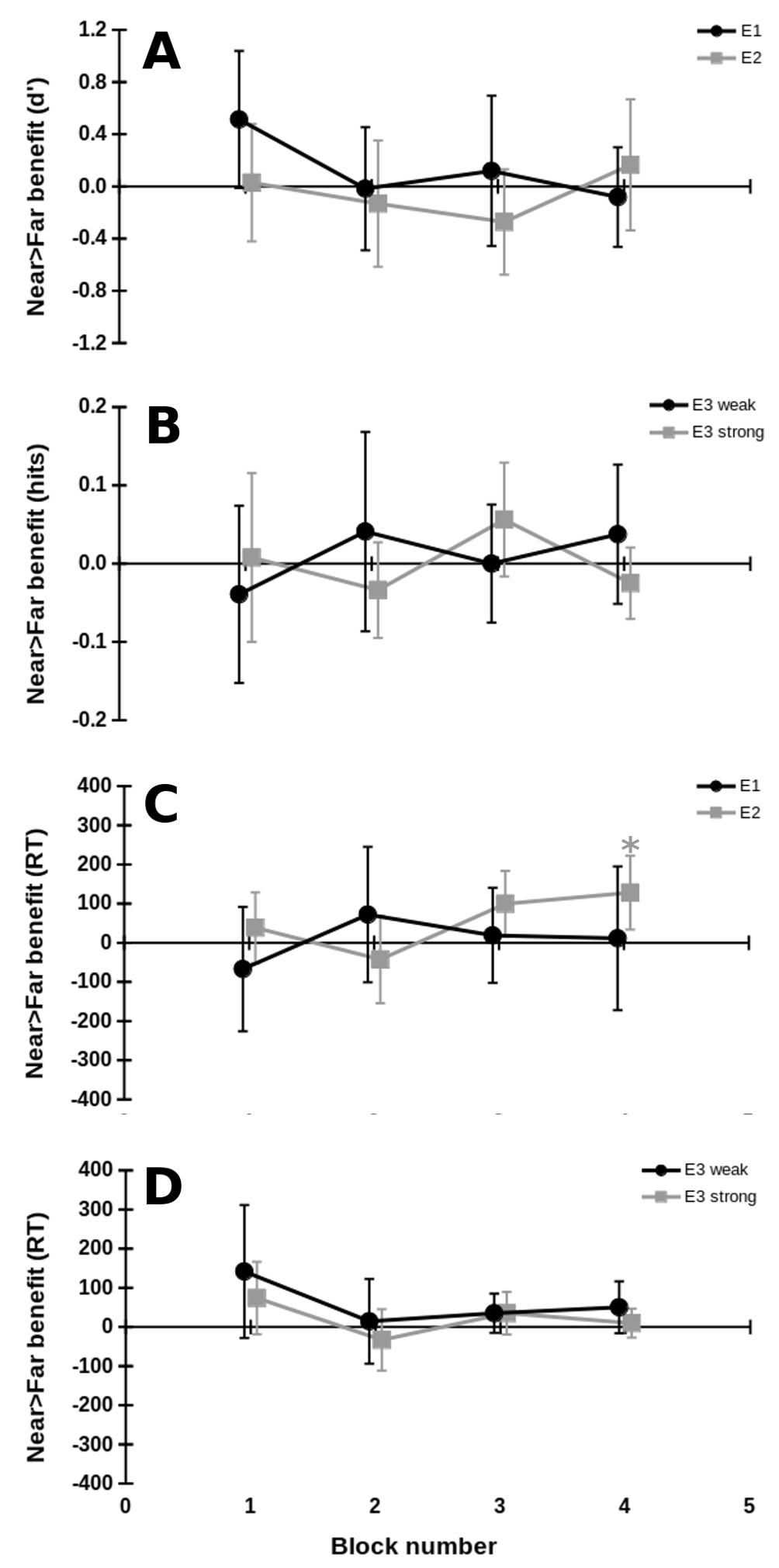


***Supplementary Figure 3****. No effect of block number on the Near>Far benefit in the current study. Data from Experiments 1-3 (E1-3) were re-analysed with block as a variable. Data are means±95% confidence intervals. Correcting for 4 comparisons against zero in each experiment and dependent variable (alpha=.0125), only one of the blocks produced data showing a significant Near>Far benefit (Experiment 2, block 4, RT, panel C).* ***A****. Experiments 1 & 2, d-prime.* ***B****. Experiment 3 hits.* ***C****. Experiments 1 & 2, reaction time (RT), * p<.0125,* ***D****. Experiment 3, reaction time (RT).*

*Statistical analysis showed no effect of block in Experiment 1 (d-prime, F(3,39)=1.31, p=.29; RT, F(3,39)=0.51, p=.68), Experiment 2 (d-prime, F(3,45)=0.87, p=.46; RT, F(3,45)=2.71, p=.06), or Experiment 3 (hits weak target, F(3,45)=0.71, p=.55; hits strong target, F(3,45)=1.29, p=.29; RT weak target, F(3,45)=1.60, p=.20; RT strong target, F(3,45)=2.52, p=.07).*

***Supplementary Table 4: Effects of auditory stimuli near (~30cm) and far from (~100cm) the trunk***

| **Study, experiment, condition** | **Near** | | **Far** | | **Far-Near effect** | |
| --- | --- | --- | --- | --- | --- | --- |
|  | **~30cm** | **RT (ms)** | **~100cm** | **RT (ms)** | **ms** | **ms/m** |
| Serino et al. 2015 E1, looming | D2, 24cm | -56 | D2, 100cm | +15 | 71 | 93 |
| Serino et al. 2015 E4. looming | D2, 24cm | -48 | D2, 100cm | +3 | 51 | 67 |
| Serino et al. 2015 E6, congruent | D2, 37cm | -50 | D4, 101cm | -23 | 27 | 42 |
| Galli et al. 2015 E1 pre | B/F1, 28.5cm | 305 | B/F3-4, 100cm | 303 | -2 | -3 |
| Galli et al. 2015 E2 pre | B/F1, 28.5cm | 289 | B/F3-4, 100cm | 294 | 5 | 5 |
| Galli et al. 2015 E3 pre | B/F1, 28.5cm | 285 | B/F3-4, 100cm | 296 | 11 | 15 |
| Noel et al. 2015a E1, standing | D1, 33cm | -27 | D3, 100cm | -12 | 15 | 22 |
| Noel et al. 2015a E2, standing | D1, 33cm | -33 | D3, 100cm | -4 | 29 | 43 |
| Noel et al. 2015b E1, asynchrony | D2, 30cm | 330 | D6, 90cm | 396 | 66 | 110 |
| Noel et al. 2015b E2, asynchrony | D2, 30cm | 317 | D6, 90cm | 397 | 80 | 133 |
| **Across-study mean±SD** | | | | | **35±30** | **53±47** |

E: Experiment; pre: Baseline testing before the intervention; D: Distance; B: Back; F: Front; RT: Reaction time or reaction time difference from baseline; ms: milliseconds; m: metres; SD: standard deviation

Converting the 53ms/m into a prediction for a 70cm difference (i.e., 30 vs 100cm) results in a predicted 37±33ms difference between near and far auditory stimuli both presented *within* trunk-centred peripersonal space.

**Supplementary Experiment 5: Can participants perform the 1-interval discrimination task used in Experiments 1 and 2?**

During the review process, it was suggested that the reason participants could not perform the tactile discrimination task used in Experiments 1 and 2 was due to the type of stimuli used (i.e., white noise), whereas in Serino et al. (2007), the target was an electrical stimulus. Unfortunately, our laboratory does not have the necessary equipment to present electrical stimuli in a controlled way. We can not, therefore, give any answer about potential differences between electrical and vibrotactile stimuli in this task.

To address this concern, we set up an apparatus similar to that used in the original experiments (which were conducted with different equipment in the University of Reading in 2013-14), this time in the University of Nottingham in 2019. While the vibrotactile stimulators were of the same type, the original computers and peripheral hardware used to generate and amplify the target stimuli were not available, so new hardware was used. We cannot, therefore, be sure that the intensities of stimuli used in this experiment are comparable with those of Experiments 1 to 4.

Our aim here was only to test whether it was *possible* for participants to perform the vibrotactile discrimination task used in Experiments 1 and 2. The first author (NPH) and two other participants performed two 1-hour sessions in which they detected and discriminated between vibrotactile stimuli under several experimental conditions. These conditions included the experimental design: 1-interval (1-IFC) vs. 2-interval (2-IFC), the task: detection vs. discrimination, and the type of auditory stimulus: continuous background noise vs. discrete pulses of noise as used in Experiments 1-4. Training was given until each participant could reach a 90% detection threshold without the QUEST algorithm reaching the ceiling (i.e., performance consistently worse than 90% correct).

First, participants performed a 2-IFC and 1-IFC detection tasks once with continuous and once with discrete white noise. In all four tasks, all three participants reached a threshold. For participant 1 (NPH, highly-trained on the task), stimulus intensity at threshold was 0.19-0.236 (arbitrary units), for participant 2 (well-trained) they were 0.973-3.42 A.U., while for participant 3 (untrained) they were 5.11-9.19 A.U. This 50-fold range in detection threshold across participants and dependent on training is not unusual for these tasks and stimuli.

Second, participants performed 2-IFC and 1-IFC intensity discrimination tasks once with continuous and once with discrete white noise. In the 2-IFC task, one interval always had a stimulus with intensity at the 90% detection threshold, the other interval had a stimulus 1.5x stronger than the 90% threshold. Participants responded to which interval contained the strong target, as in Experiments 1-2. In the 1-IFC task, each interval had either a strong or a weak stimulus and participants responded according to which was presented. Performance on these tasks was quite poor. With 44 trials, participants needed to get 27/44 trials correct in order to perform better than chance (binomial test). Participant #1 reached this level for all four tasks (70-84% correct), and participants 2 & 3 reached this level in the 2-IFC task with continuous noise (both 61%), but not in the others (continuous 1-IFC: 41, 48%; discrete 1-IFC: 52, 55%; discrete 2-IFC: 59, 57%).

The conclusion from these three participants is that the discrimination task is possible, but seems to be very difficult, and requires training to achieve consistently better-than-chance performance. It is not known whether this difficulty is due to the task, the levels of stimulus intensity used, or the type of stimuli chosen.

In a separate session, Participant #1 also performed 1-IFC discrimination with continuous and discrete noise at 7 different levels of target intensity relative to the 90% threshold (1.2, 1.4, 1.6, 1.8, 2.0, 2.2, and 2.4 times the 90% detection threshold). In all 14 blocks, Participant #1 performed better than chance, and performance improved with an increasing ‘strong’ target intensity relative to the ‘weak’ target. Performance seemed to be better overall with the discrete than with continuous white noise bursts (Supplementary Figure 4).


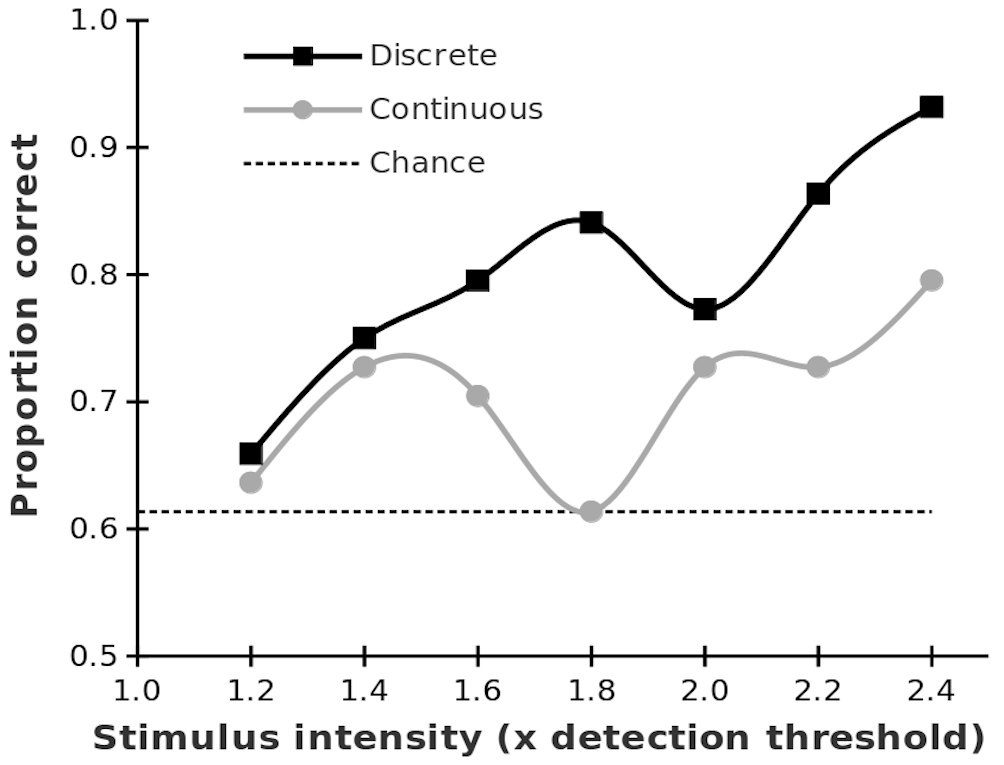


*Supplementary Figure 4: Performance on the 1-IFC intensity discrimination task used in Experiments 1-3, by a single, highly-trained observer (the author, NPH). The stimulus intensities used in Experiments 1-3 were at 1.0 (weak) and 1.5 (strong) on the x-axis. The data show the proportion correct discriminations between weak and strong stimuli in the 1-IFC task with continuous (grey) or discrete bursts (black) of white noise. The broken horizontal line shows the chance level for an individual block of trials. While it was possible* for a well-trained observer to perform the task consistently better than chance (all blocks individually better than chance, p<.05), it was too difficult for less-well-trained participants.
